# Modified TMV Particles as Beneficial Scaffolds to Present Sensor Enzymes

**DOI:** 10.3389/fpls.2015.01137

**Published:** 2015-12-24

**Authors:** Claudia Koch, Katrin Wabbel, Fabian J. Eber, Peter Krolla-Sidenstein, Carlos Azucena, Hartmut Gliemann, Sabine Eiben, Fania Geiger, Christina Wege

**Affiliations:** ^1^Department of Molecular Biology and Plant Virology, Institute of Biomaterials and Biomolecular Systems, University of StuttgartStuttgart, Germany; ^2^Chemistry of Oxydic and Organic Interfaces, Karlsruhe Institute of Technology, Institute of Functional InterfacesKarlsruhe, Germany; ^3^Department of New Materials and Biosystems, Max-Planck-Institute for Intelligent SystemsStuttgart, Germany

**Keywords:** tobacco mosaic virus (TMV), biotemplate, scaffold, glucose sensor, enzyme cascade system, enzyme shelf-life, reusability, surface immobilization

## Abstract

*Tobacco mosaic virus* (TMV) is a robust nanotubular nucleoprotein scaffold increasingly employed for the high density presentation of functional molecules such as peptides, fluorescent dyes, and antibodies. We report on its use as advantageous carrier for sensor enzymes. A TMV mutant with a cysteine residue exposed on every coat protein (CP) subunit (TMV_Cys_) enabled the coupling of bifunctional maleimide-polyethylene glycol (PEG)-biotin linkers (TMV_Cys_/Bio). Its surface was equipped with two streptavidin [SA]-conjugated enzymes: glucose oxidase ([SA]-GOx) and horseradish peroxidase ([SA]-HRP). At least 50% of the CPs were decorated with a linker molecule, and all thereof with active enzymes. Upon use as adapter scaffolds in conventional “high-binding” microtiter plates, TMV sticks allowed the immobilization of up to 45-fold higher catalytic activities than control samples with the same input of enzymes. Moreover, they increased storage stability and reusability in relation to enzymes applied directly to microtiter plate wells. The functionalized TMV adsorbed to solid supports showed a homogeneous distribution of the conjugated enzymes and structural integrity of the nanorods upon transmission electron and atomic force microscopy. The high surface-increase and steric accessibility of the viral scaffolds in combination with the biochemical environment provided by the plant viral coat may explain the beneficial effects. TMV can, thus, serve as a favorable multivalent nanoscale platform for the ordered presentation of bioactive proteins.

## Introduction

Biological scaffolds have been used for the spatially precise immobilization and presentation of organic or inorganic materials and functional molecules increasingly during the past decades, due to their regular shapes, multivalence on the nanometer scale and self-assembly capabilities. They comprise, amongst others, DNA origami structures (e.g., Maune et al., 2010; Saccà and Niemeyer, 2012, and references therein; Said et al., 2013), self-assembled lipid microstructures (e.g., Zhou and Shimizu, 2008; Namiki et al., 2011), bacterial surface (S-) layer proteins (reviewed by Sleytr et al., 2007, 2014), clathrin- and apoferritin-derived protein nanocages (e.g., Zhang and Knez, 2012; Schoen et al., 2013; Huggins et al., 2014), protein origami (Lai et al., 2013), bacteriophages (e.g., Singh et al., 2006; Petrenko, 2008; Mao et al., 2009; O'Neil et al., 2011; Cardinale et al., 2012, and references therein), and viruses (for fundamental studies and recent reviews refer to Douglas and Young, 1998; Singh et al., 2006; Aniagyei et al., 2008; Young et al., 2008; Lee et al., 2009; Liu et al., 2012b; Pushko et al., 2013; Glasgow and Tullman-Ercek, 2014; Khudyakov and Pumpens, 2015, and numerous references therein; Putri et al., 2015).

Viruses exhibit a highly ordered 3D nanoscale architecture assembled from up to thousand copies of one or few coat protein (CP) types, which protect the viral nucleic acid genomes. Viral capsids may be arranged in a spherical (quasi-icosahedral) or rod-shaped (helical) manner, in the case of most plant-infecting viruses without a lipid envelope (King et al., 2012). These structural features result in well-defined interior and exterior protein surfaces, precise spatial localization of chemically reactive groups, and in uniformity on the nanometer scale. Furthermore, several virions are extremely stable, can be produced in large quantities and modified genetically as well as chemically (for recent reviews, see Soto and Ratna, 2010; Liu et al., 2012b; Bittner et al., 2013; Bernard and Francis, 2014; Culver et al., 2015). Since they are highly promising building blocks to construct novel nanostructured materials, plant viruses have served as scaffolds for a wide range of functional molecules such as reporter dyes (e.g., Cruz et al., 1996; Gillitzer et al., 2002; Lewis et al., 2006; Martin et al., 2006), antigens for vaccination purposes as reviewed in detail (e.g., Chackerian, 2007; Crisci et al., 2012; Kushnir et al., 2012), antibodies as tracers in immunoassays (Sapsford et al., 2006) or immunoadsorbents (Werner et al., 2006), medical imaging reagents or drugs (with numerous examples described in Yildiz et al., 2011; Khudyakov and Pumpens, 2015), and a plentitude of inorganic and synthetic compounds to fabricate technically applicable hybrid materials and devices with novel physical and chemical properties (reviewed in Lee et al., 2012b; Bittner et al., 2013; Li and Wang, 2014; Love et al., 2014; Culver et al., 2015). An emerging field is the immobilization of proteins conferring complex functionalities, including e.g., receptor- or hapten-binding modules and enzymes (e.g., Chatterji et al., 2004; Carette et al., 2007; Soto et al., 2009; Szuchmacher Blum et al., 2011; Aljabali et al., 2012; Cardinale et al., 2012, and references therein; Pille et al., 2013).

Main objects in this context are the spherical *cowpea mosaic virus* (CPMV), *cowpea chlorotic mottle virus* and *brome mosaic virus*, the filamentous *potato viru*s *X* (PVX), and the rod-shaped *tobacco mosaic virus* (TMV) (Singh et al., 2006; Lee et al., 2009; Mao et al., 2009; Liu et al., 2012b; Bittner et al., 2013; Glasgow and Tullman-Ercek, 2014; Culver et al., 2015). The presentation of functional proteins on the viral surfaces revealed well-preserved biological activities (e.g., Chatterji et al., 2004; Carette et al., 2007; Frolova et al., 2010; Szuchmacher Blum et al., 2011; Pille et al., 2013). However, depending on the viral backbones, the proteins of interest and the respective coupling strategies, occupation rates of the carrier templates varied substantially. A rare example of a combination of cooperating enzymes on a plant viral scaffold has been analyzed in detail for CPMV, with glucose oxidase (GOx) and horseradish peroxidase (HRP) displayed on the exterior of the capsids via carbohydrate-carboxylate-mediated covalent attachment (Aljabali et al., 2012). Both enzymes were shown to retain their activities, but could be immobilized in low amounts only, probably due to steric constraints.

Here, we employed complete TMV as well as lower-order TMV CP aggregates as scaffolds for the immobilization of the same enzyme system, with some improvements such as a linker-mediated conjugation to efficiently address the outer surface of chemically compatible virus variants (Geiger et al., 2013). The length of the bifunctional cross-linkers should provide more degrees of freedom for a dense decoration of the viral assemblies. The rigid TMV rod is especially robust and allows a stable display of various heterologous compounds (Alonso et al., 2013; Bittner et al., 2013; Love et al., 2014; Culver et al., 2015). It consists of 2130 identical, helically arranged CP subunits encapsidating a positive-sense single-stranded viral RNA genome of 6395 nucleotides sandwiched between the CP helix (Caspar, 1963; Namba et al., 1989). CPs are also able to self-assemble into small oligomers (“A-proteins”), ring-shaped two-layer structures (“disks” or “20S-aggregates”), and stacked disks of different multimerization grades in the absence of compatible RNA, depending on the environmental conditions (Butler, 1971, 1999). The complete TMV rod has a length of 300 nm, an outer diameter of 18 nm and an inner channel of 4 nm width (Zaitlin, 2000). TMV-like particles with altered lengths or even kinked or branched shapes, freely suspended, or immobilized at one end on solid supports, can be obtained by *in vitro* assembly using RNA constructs with the viral origin of assembly sequence (Mueller et al., 2011; Azucena et al., 2012; Eber et al., 2013, 2015; Kadri et al., 2013; Rego et al., 2013). Moreover, different TMV CP variants may be combined in individual particles, either mixed, or arranged into longitudinal rod domains (Geiger et al., 2013; Eiben et al., 2014). This flexibility in adjustable shape and composition makes TMV derivatives particularly versatile biotemplates.

Correspondingly, TMV-like particles have been applied for the fabrication of nanowires by metallization of their inner or outer surfaces (Douglas and Young, 1998; Knez et al., 2003, 2004; Lee et al., 2006; Balci et al., 2009; Lewis et al., 2010; Manocchi et al., 2010; Kadri et al., 2011; Zhou et al., 2015, and further studies), as stabilizer for magnetorheological ferrofluids (Wu et al., 2010), mineralization-guiding templates yielding e.g., components for electronic or energy conversion devices (e.g., Shenton et al., 1999; Lee et al., 2006; Nam et al., 2006; Royston et al., 2008, 2009; Atanasova et al., 2011, 2015; Chen et al., 2011; Witus and Francis, 2011; Chiang et al., 2012; Altintoprak et al., 2015), scaffolds for the presentation of fluorescent dyes or contrast agents, including advantageous nanoparticles for targeting and intravital imaging purposes (e.g., Demir and Stowell, 2002; Schlick et al., 2005; Smith et al., 2006; Lewis et al., 2010; Wen et al., 2012, 2015; Bruckman et al., 2015; Shukla et al., 2015), as antigens for vaccine applications (e.g., Turpen et al., 1995; Smith et al., 2006; McCormick and Palmer, 2008; Karpova et al., 2012; Banik et al., 2015), and cell or tissue-culture supports and additives (e.g., Lee et al., 2012a; Luckanagul et al., 2015).

Here we report a dense immobilization of active enzymes on TMV templates for the first time. A cysteine-modified TMV variant (TMV_Cys_; S3C) nearby the CP N-terminus described previously (Geiger et al., 2013) (resembling TMV mutants described by Yi et al., 2005; Smith et al., 2006), with more than 2000 addressable thiol groups, was coupled with bifunctional maleimide-polyethylene glycol (PEG)-biotin linkers yielding TMV_Cys_/Bio. This scaffold was used to present streptavidin-conjugated GOx and HRP, a well-established two-enzyme system (Woolridge et al., 1986; Bateman and Evans, 1995; Aljabali et al., 2012). GOx is a globular dimeric protein that catalyzes the oxidation of glucose to D-glucono-1,5-lactone, with molecular oxygen as electron acceptor. It produces hydrogen peroxide (Hecht et al., 1993), which is a substrate for HRP, a monomeric enzyme which reduces hydrogen peroxide to water and may concomitantly convert chromogenic substrates [e.g., TMB: 3,3′,5,5′-tetramethylbenzidine, DAB: 3,3′-diaminobenzidine, ABTS: 2,2′-azino-bis(3-ethylbenzothiazoline-6-sulphonic acid)] into colored products for detection purposes (Azevedo et al., 2003). The following data show that TMV sticks allow an efficient immobilization of active enzymes and exert positive effects on the biomolecules' stability in comparison to other supports or adapters tested.

## Materials and methods

All (bio-) chemicals were purchased from Roth (Karlsruhe, Germany) and used according to the manufacturer's protocols unless otherwise stated. UV absorption spectra of proteins, nucleic acids, or virus particles were determined with Nanodrop instruments (Peqlab, Erlangen, Germany). Images were evaluated with Image J (Rasband, 1997–2010), graphs and diagrams generated using Inkscape (Software Freedom Conservancy, Brooklyn, NY) and GraphPad Prism 4 (GraphPad Software Inc., San Diego, CA, USA). Table 1 lists the abbreviations of the major biological or biochemical building blocks combined in the different enzyme-exposing adapter constructs used in this study.

**Table 1:** **Abbreviations of biological/biochemical building blocks**.

**Abbreviation**	**Description**
TMV	*Tobacco mosaic virus* (nanorod particles)
TMV_Cys_	TMV particles containing S3C-mutant coat proteins
TMV_Cys_/Bio	TMV_Cys_ equipped with [maleimide-]-PEG_11_-biotin linkers
CP	Coat protein
CP_Cys_/Bio	Coat protein of TMV_Cys_, equipped with biotin linkers
[SA]	Streptavidin
GOx	Glucose oxidase
[SA]-GOx	Streptavidin-conjugated GOx
HRP	Horseradish peroxidase
[SA]-HRP	Streptavidin-conjugated HRP
Biotin-	Maleimide-PEG_11_-biotin linker (in compound forms)

### Virus and CP preparation

TMV_WT_ or TMV_Cys_ particles (Geiger et al., 2013) were isolated from systemically infected *N. tabacum* “Samsun” nn plants according to Chapman (1998), involving 1-butanol extraction and PEG precipitation followed by ultracentrifugation (UC). CP_WT_ or CP_Cys_ were purified from virions using an acetic acid-based method (Fraenkel-Conrat, 1957) followed by dialysis against ultrapure water (ddH_2_O, 18.3 MΩ cm, purified by a membraPure system, Aquintus, Bodenheim, Germany). The purity and concentration of TMV and CP preparations were determined spectrophotometrically. The resulting solutions (5–10 mg/ml) were stored at 4°C in 10 mM sodium potassium phosphate buffer (SPP) pH 7.0.

### Biotinylation of TMV_Cys_

Biotin was covalently conjugated to the cysteine residues of TMV_Cys_ using a biotin linker (EZ-Link® Maleimide-PEG_11_-Biotin; Thermo Scientific, Rockford, IL) in 22-fold molar excess over CP_Cys_ coupling sites in 10 mM SPP pH 7.0 for 16 h with gentle agitation at room temperature (RT). TMV_WT_ was used as a control under equal labeling conditions. Unbound maleimide-biotin linker molecules were removed by PEG precipitation in exploratory experiments, or by ultracentrifugation resulting in improved recovery (UC; 134,000 × g, 4°C, 2 h; comparative data not shown). Pellets were resuspended in 10 mM SPP pH 7.0. Removal of free linkers was confirmed by dot blot analysis in combination with streptavidin-conjugated alkaline phosphatase ([SA]-AP)-mediated biotin detection (Supplementary Figure 1). Resuspended TMV_Cys_/Bio particles in SPP were further analyzed by 15% SDS-PAGE (Laemmli, 1970). Samples (final concentration (f.c.): 1 μg TMV/10 μl) were mixed with loading buffer (f.c.: 50 mM Tris-HCl pH 6.8, 2% (w/v) SDS, 0.1% (w/v) bromophenol blue, 10% (v/v) glycerol, 100 mM dithiothreitol) and heated for 5 min at 95°C. Gels were stained with Coomassie Brilliant Blue R250 (Serva Electrophoresis, Heidelberg, Germany). Coupling efficiency of linker to CP was determined densitometrically via image evaluation software Image J (Rasband, 1997–2010). Free CP_Cys_/Bio protein solutions were obtained from TMV_Cys_/Bio particles by acetic acid treatment and dialysis as described above.

### Coupling of streptavidin-conjugated enzymes to TMV_Cys_/Bio nanorods in solution and enzyme activity determination

TMV_Cys_/Bio were coupled to streptavidin-conjugated GOx ([SA]-GOx) and HRP ([SA]-HRP) (SDT, Baesweiler, Germany; Streptavidin-HRP #SM1C, Streptavidin-Glucose oxidase #SG1, both with no more than one enzyme per SA according to the supplier) in a molar ratio of [SA]-GOx:[SA]-HRP of 14 to 1. This ratio was deduced from preliminary experiments to determine the catalytic efficiencies of [SA]-enzymes (for details, see Supplementary Section 4), which resulted in k_cat_values of 157.68 1/s for [SA]-HRP, and 9.33 1/s for [SA]-GOx, indicating that the [SA]-HRP used had a ~17-fold higher activity than the rate-limiting [SA]-GOx. To improve the accuracy of measurements by slowing down the reaction rates, a molar ratio of 14 to 1 of [SA]-GOx to [SA]-HRP was employed in all investigations described in detail below. For installing the enzymes on TMV scaffolds, the [SA]-GOx/[SA]-HRP mixture (10 μg) was diluted together with TMV_Cys_/Bio (0.8 μg) in 50 μl 10 mM SPP pH 7.0, corresponding to one enzyme molecule per CP subunit. The mixture was incubated for 3 h at RT and unbound [SA]-GOx and [SA]-HRP molecules were separated by UC (134,000 × g, 4°C, 2 h). The supernatant was kept on ice and the pellet resuspended in 50 μl 10 mM SPP pH 7.0. For comparison, a sample containing the GOx/HRP mixture only was processed in parallel. Samples were then diluted 1:100 in 10 mM SPP pH 7.0, and 50 μl aliquots were applied to microplates (96-well clear polystyrene; Greiner Bio-One, Frickenhausen, Germany). One hundred and fifty microliter substrate mixture (f.c.: 5 mM ABTS, 50 mM NaOAc, 100 mM glucose) per well were added and the activities of TMV-bound and unbound enzymes were determined spectrophotometrically at 405 nm for 30 min (SpectrafluorPlus, TECAN). Product concentrations were calculated from absorption values using the Lambert-Beer law $c=\frac{A}{d\;\cdot \varepsilon_{405}}$ with *A* = detected absorption at 405 nm, *d* = filling level of well (0.625 cm), ε_405_nm = $\frac{36.8}{\text{mM}\cdot \text{cm}}$ (extinction coefficient of ABTS^*^ according to Childs and Bardsley, 1975, and the supplier), and plotted vs. time. Turnover rates *v* of ABTS into the corresponding radical (ABTS^*^) were inferred from the slopes of the linear sections (y = mx + c; *r*^2^ was found to be between 0.96 and 0.99 consistently). They are proportional to the glucose turnover which equals 50% of the molar ABTS conversion (underlying reaction cascade: $\text{Glucose}\frac{[\text{SA}]-\text{GOx}}{}\text{​​​}>\text{Gluconolactone}+{\text{H}}_{2}{\text{O}}_{2};$; $2\text{ABTS}+{\text{H}}_{2}{\text{O}}_{2}\frac{[\text{SA}]-\text{HRP}}{}>2{\text{ABTS}}^{*}+2{\text{H}}_{2}\text{O})$. All reactions were carried out as technical triplicates, experiments were repeated at least three times.

### Enzyme coupling on immobilized adapters (TMV_Cys_/Bio, CP_Cys_/Bio or maleimide-biotin linker) and determination of catalytic activities

Immobilization of biotinylated TMV, CP, or linker molecules (collectively referred to as “adapters” in the following) and enzyme coupling reactions were performed in microtiter plates (clear F-bottom 96-well polystyrene high binding microlon®; No. 655061, Greiner Bio-One, Frickenhausen, Germany). Specifically, TMV_Cys_/Bio nanorods or CP_Cys_/Bio aggregates, or maleimide-biotin linkers in equivalent amounts serving as adapters between plates and [SA]-enzymes, were diluted in 50 μl binding buffer (10 mM SPP pH 7.85 supplemented with 137 mM NaCl) and applied to the wells overnight at 4°C. The equivalence of amounts for maleimide-biotin linkers was determined in relation to the number of biotinylated CPs in the TMV_Cys_/Bio nanorods assuming 50% biotinylation efficiency as deduced from the analyses described above. This binding step was followed by three consecutive washings at RT for 5 min with 1x PBS-T (PBS, 0.05% Tween-20). To reduce non-specific enzyme binding, wells were blocked with 200 μl 2% BSA (w/v) in 1x PBS (Green and Sambrook, 2012) for 1.25 h at RT after adapter immobilization, and washed as before. The enzyme mix ([SA]-GOx:[SA]-HRP = 14:1 as described above; in total 0.9 μg [SA]-enzymes per well) was added for 3 h. Following three washings, ABTS turnover rates were determined after adding 200 μl substrate mixture as described in the previous paragraph.

### Reusability and storage stability

The adapter constructs, i.e., TMV_Cys_/Bio nanorods, CP_Cys_/Bio aggregates or equivalent amounts of biotin linkers, were applied to the high binding microtiter plate wells. Initial catalytic activities (first use) were determined as described above. Afterwards, the adapter-immobilized enzymes were washed three times with 1x PBS-T and stored in 10 mM SPP pH 7.0 at RT before repeated hourly measurements of ABTS turnover rates with fresh substrate mixture, and intermittent storage in SPP, over a period of 7 h in total. Initial referential activities (*h* = 0) for each adapter-enzyme combination were set to 100% and the percentages of remaining activities were monitored.

In an expanded setting of the experiment, samples were stored in 10 mM SPP pH 7.0 at 4°C for 3 weeks and their activities serially determined at extended intervals (initial referential activity = day 0). To investigate possible influences of the adapters on the long-term storage stability of non-activated enzymes, samples prepared simultaneously were measured at distinct time points after storage without prior use. In this setup, 8-well strip plates (clear F-bottom 12 × F8 strip plates mounted in frame, polystyrene high binding microlon® No. 762071, Greiner Bio-One, Frickenhausen, Germany) were employed, to allow the analysis of separate well rows kept continuously at 4°C in 10 mM SPP pH 7.0 before use.

### Atomic force microscopy (AFM)

Following adapter immobilization and enzyme coupling, the well-bottoms of high binding plates were punched with the help of a self-made device, rinsed with ddH_2_O, dried in a nitrogen stream and used for AFM analysis (MFP-3D BIO; Asylum Research: Oxford Instruments, Santa Barbara, CA, USA) operating in air at RT in intermittent contact mode, using a silicon cantilever (AC240, Olympus) with a typical force constant of 2 N/m and a resonance frequency of 70 kHz.

### Transmission electron microscopy (TEM)

Samples of TMV_Cys_, TMV_Cys_/Bio, or TMV_Cys_/Bio/[SA]-enzyme complexes (0.2 μg/μl in 10 mM SPP pH 7.0) were adsorbed for 10 min to Formvar®-coated and carbon-sputtered 400 mesh copper grids, washed three times for 1 min on drops of ddH_2_O and negatively stained using three drops of uranyl acetate (UAc) supplemented with bacitracin (2% (w/v) UAc; 250 mg/ml bacitracin), with 1 min incubation on each drop. TEM (Tecnai G2 Sphera Fei TEM; Fei, Eindhoven, Netherlands) was carried out at 120 kV using a 16 megapixel camera (TemCam-F416 (4k × 4k), TVIPS, Gauting, Germany).

## Results

First, catalytic activities of adapter-bound enzyme conjugates (i.e., [SA]-GOx and [SA]-HRP displayed on TMV_Cys_/Bio sticks, CP_Cys_/Bio aggregates, or biotin linker molecules, respectively) adsorbed to solid microtiter plate supports were compared to those of the same enzyme conjugates bound to the plates without any adapters (“free”). Subsequent tests investigated putative advantages of the TMV-based adapter systems over conventional immobilization strategies, with respect to the enzymes' stability upon repetitive uses and extended storage periods.

### TMV_Cys_ biotinylation and coupling of GOx and HRP streptavidin [SA]-conjugates

**Figure 1:**
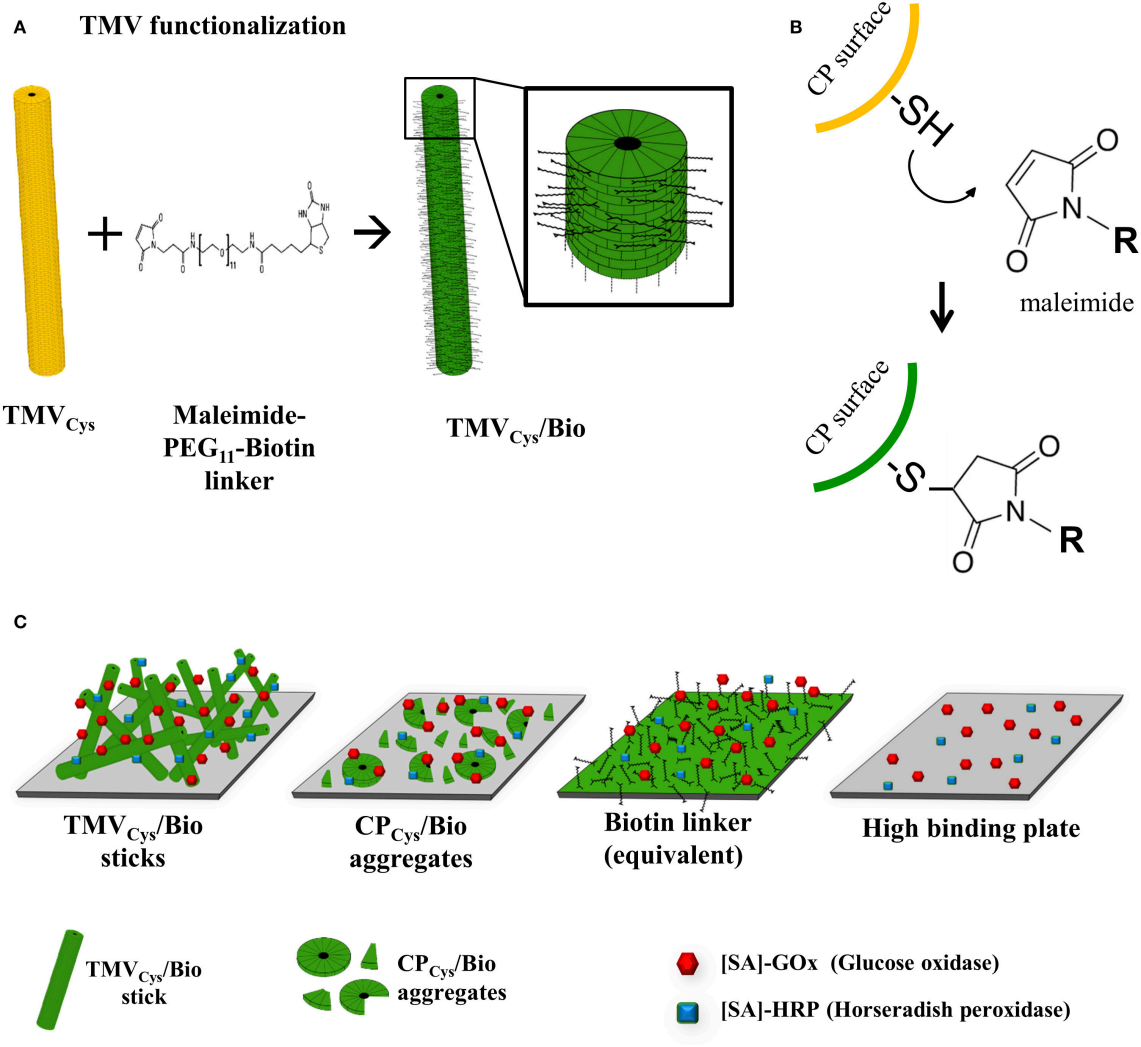
**Functionalization of TMV and immobilization strategy for coupling enzymes to viral adapters. (A)** Schematic drawing of TMV functionalization stages. Bifunctional thiol-reactive maleimide-PEG_11_-biotin linker molecules (black) were coupled to the cysteine residues on the surface of TMV_Cys_ (yellow), resulting in biotinylated TMV scaffolds (TMV_Cys_/Bio, green). The magnified TMV_Cys_/Bio section illustrates coupled linker molecules on every second coat protein, reflecting the biotinylation efficiency of 50% achieved in this study. **(B)** Reaction scheme of the specific covalent conjugation of a maleimide-activated linker to a thiol (i.e., sulfhydryl) function on the surface of a TMV_Cys_ CP, forming a stable non-reversible thioether linkage. (R) residue of the maleimide-activated cross-linker, in our case a PEG_11_-spacer terminated by biotin. **(C)** Cartoon of immobilized adapter types employed for enzyme coupling. From left to right: TMV_Cys_/Bio sticks, CP_Cys_/Bio aggregates, equivalent amounts of biotin linkers, and the untreated high binding 96-well plate used as solid support for immobilization.

**Figure 2:**
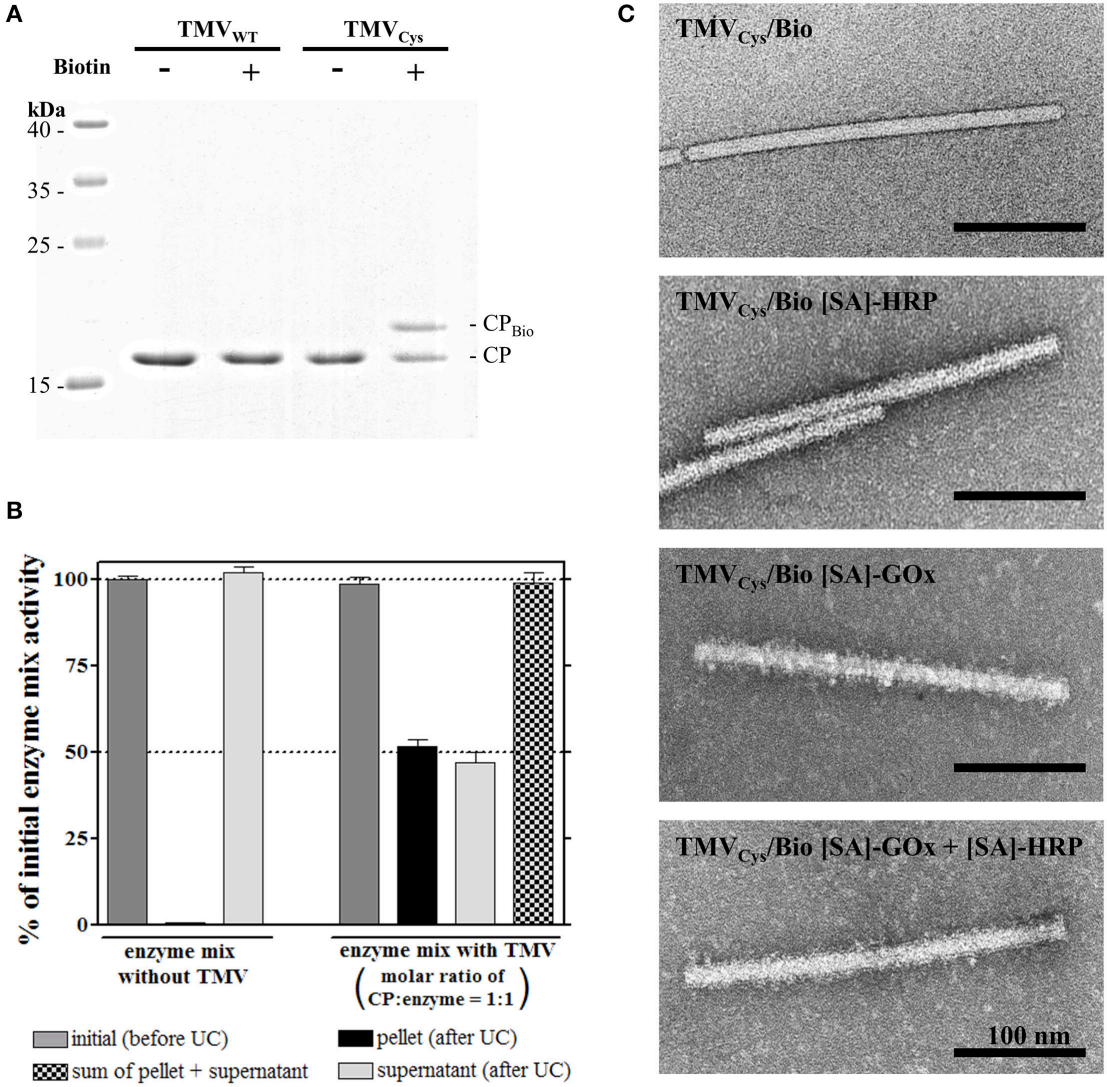
**Functionalization of TMV_**Cys**_ particles with maleimide-PEG_**11**_-biotin linker and coupling of streptavidin [SA]-tagged enzymes to TMV_**Cys**_/Bio. (A)** Selective coupling of maleimide-PEG_11_-biotin (Biotin) to TMV_Cys_. Control reactions contained TMV_WT_ or were performed in the absence of linker molecules, as indicated above the image. Samples were denatured and subjected to electrophoresis in a 15% SDS-PA gel. CP-containing bands were stained with Coomassie Brilliant Blue R250. A coupling efficiency of approximately 50% was achieved, as confirmed by image evaluation (software ImageJ). Molecular weights of marker proteins are indicated. **(B)** The efficiency of enzyme coupling to TMV_Cys_/Bio sticks in solution, and its putative effects on enzyme activity were investigated using ultracentrifugation. [SA]-enzymes ([SA]-GOx/[SA]-HRP mixture) were analyzed alone, or in the presence of TMV sticks in a molar ratio of one enzyme per CP. As indicated by the corresponding columns of the histogram, partitioning of the input activity (initial activity, gray) depended on the presence of biotinylated TMV. TMV_Cys_/Bio-[SA]-enzyme complexes were found in the pellet, uncoupled [SA]-enzymes in the supernatant. In conjunction with TMV templates, 50% of the initial activity was found in each fraction, indicating successful coupling of one enzyme to virtually every CP_Bio_. As the sum of [SA]-enzyme activity was neither affected by ultracentrifugation alone as shown for the control without TMV_Cys_/Bio (left), nor by the presence of TMV sticks (right), linkage to the adapter sticks did not influence the activity of the bi-enzyme system. **(C)** TEM micrographs of functionalized TMV_Cys_ particles. Successful coupling of [SA]-enzymes ([SA]-GOx, [SA]-HRP) resulted in the particles' decoration by an additional electron-dense layer, adding a fluffy seam to the otherwise plane contour of TMV capsid. Samples were adsorbed on Formvar-coated, carbon-sputtered copper grids, and negatively stained with 2% uranyl acetate.

TMV_Cys_ particles (Geiger et al., 2013) were purified from tobacco plants and their accessible cysteine moieties biotinylated *in vitro* via a maleimide-PEG_11_-biotin linker (Figures 1A,B). Denaturing SDS-PAGE confirmed the success of the coupling reactions, as the biotin linker (922 Da) shifted the CP_Cys_ (17.6 kDa) molecules to the expected band (Figure 2A). Control samples with TMV_WT_ lacking accessible thiols proved the specific attachment (Figure 2A). A 22-fold molar excess of biotin linker relative to the viral CP_Cys_ subunits modified approximately 50% of the target sites, as determined by densitometry, and this ratio was applied further on.

To test the efficiency of enzyme coupling and to investigate the influence of the binding on the enzyme activity, TMV_Cys_/Bio was incubated with a 12-fold (w/w) excess of enzymes (blended in a molar ratio of 14 to 1 of [SA]-GOx to [SA]-HRP; see Section Coupling of Streptavidin-Conjugated Enzymes to TMV_Cys_/Bio Nanorods in Solution and Enzyme Activity Determination), i.e., a ratio of one enzyme per CP. The resulting complexes were separated from unbound [SA]-enzymes using ultracentrifugal sedimentation. Approximately 50% of the input [SA]-enzyme activity was found each in the pellet and in the supernatant. In control samples with [SA]-enzymes but without TMV_Cys_/Bio, [SA]-enzymes were neither impaired in activity nor sedimented by UC (Figure 2B). The fully preserved ABTS turnover rates showed that [SA]-enzyme binding to TMV_Cys_/Bio did not affect [SA]-GOx or [SA]-HRP catalytic activity. Since 50% of CPs carried a biotin linker, and a 1:1 ratio of CP to enzyme molecules was used, a coupling of one enzyme to virtually every biotinylated CP was achieved.

The structural integrity of TMV_Cys_/Bio without or with [SA]-enzyme conjugates was verified by TEM (Figure 2C), since most TMV nanotubes showed the expected length of 300 nm. TMV_Cys_/Bio with [SA]-enzymes installed on the linker coating, however, exhibited an increased diameter (on average 24–28 nm; compared to 18 nm for TMV_Cys_), corresponding to an additional electron dense 3–5 nm layer around the TMV scaffolds, with additional features sticking out. The originally smooth TMV surface was surrounded by a fluffy seam of conjugated enzymes, distributed largely homogenously over the whole length of the particles.

### Immobilization efficiencies of [SA]-GOx and [SA]-HRP activities with different adapters on solid supports

Whether high-density enzyme coupling to the nanobiotemplates would allow increased catalytic activities compared to those obtained after conventional (direct, adsorptive) binding to microtiter plate cavities was tested for equal input amounts of the [SA]-GOx/[SA]-HRP mixture. In standard assays, the enzymes are usually stabilized by bovine serum albumin (BSA) which competes for binding sites on the solid supports and thus limits well-loading. Furthermore, non-directional immobilization can impair enzyme functionality. Activities were, therefore, analyzed in direct comparison between different setups employing biotinylated TMV nanotubes, equivalent amounts of biotinylated TMV CP preparations, biotin-exposing chemical linkers and no adapters on the plates. In order to discriminate between selective bioaffinity binding and non-specific adhesion, non-biotinylated TMV particles with different surfaces were included in the tests.

Experiments without and with TMV_Cys_, TMV_WT_, or TMV_Lys_-loaded plates compared to TMV_Cys_/Bio nanotube adapters demonstrated the specificity and benefit of the biotin-streptavidin binding. The presence of biotin was crucial to immobilize amounts of active [SA]-enzymes sufficient for detectable substrate conversion under the conditions applied, while none of the other three TMV variants tested attached the enzymes non-specifically to a significant extent (Supplementary Figure 2).

Next, we were interested to find out if TMV nanosticks, TMV CP of lower oligomerization grade and equal amounts of biotin linker differed in immobilization efficiency upon their use as adapter scaffolds on the solid support. TMV_Cys_/Bio was either applied as intact rods, or disassembled and separated from the viral RNA to obtain a solution of free biotinylated CP aggregates (CP_Cys_/Bio) employed in a second layout. Third, equivalent amounts of maleimide-PEG-biotin linker were placed into the microtiter plate wells. In parallel, [SA]-enzymes (“free”) were immobilized directly on the untreated plate (Figure 1C).

**Figure 3:**
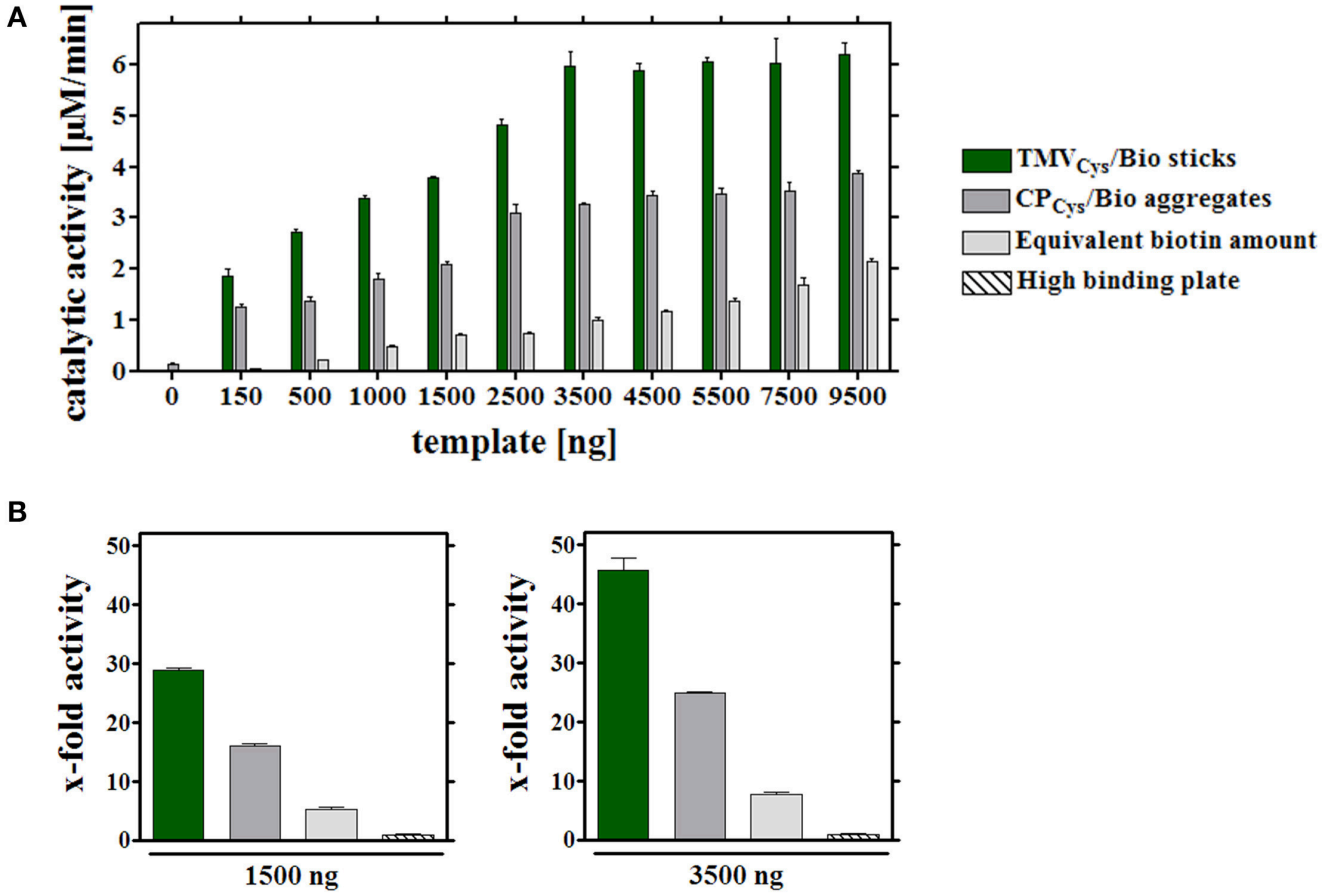
**Catalytic activities of enzymes coupled to different immobilized adapters. (A)** Different carrier scaffolds (adapters) immobilized on a solid support (in a high binding microtiter plate) were analyzed for the ABTS turnover rates achieved with equal input amounts of enzymes. To investigate their binding capacity, wells were functionalized with different quantities (between 0 and 9500 ng) TMV_Cys_/Bio sticks, CP_Cys_/Bio aggregates or the equivalent amounts of biotin linkers. 0.9 μg [SA]-enzymes per well were added and coupling to the biotin, or the non-treated plate support, allowed. Catalytic activities of immobilized [SA]-enzymes were determined spectroscopically using ABTS as substrate, and depicted in the histogram for the different assay layouts, as specified in the legend. The experiment indicated a maximum ABTS turnover rate upon application of 3.5 μg TMV_Cys_/Bio adapter sticks per well. **(B)** Percental turnover rates of ABTS reached with equal amounts of [SA]-enzyme conjugates and different adapters, normalized to the turnover achieved on bare plate support. Left: application of 1500 ng adapters (or molar equivalent of linker) per well (non-saturated conditions), right: 3500 ng adapters (or molar linker equivalent) per well, respectively, as indicated. For details, refer to text.

The binding capacities of the wells were determined by applying variable quantities of all adapter types and, after thorough washing, a constant enzyme amount and concentration in every layout. Following removal of unbound [SA]-GOx/[SA]-HRP, absolute ABTS turnover rates were calculated after spectrophotometry of the increasing ABTS^*^ concentrations as a function of time. For all equivalent biotin concentrations exposed on the different adapters, enzyme activities surpassed considerably those achieved on bare plate supports (Figure 3A). The highest substrate conversion rates were obtained with enzyme-decorated TMV_Cys_/Bio sticks. The respective turnover rates scaled with the amount of enzyme-loaded TMV templates up to 3.5 μg TMV sticks per well. It reached a maximum of roughly 6 μM ABTS conversion per min, indicating saturation of the plate cavities at this point. Using [SA]-enzyme-equipped CP_Cys_/Bio, it was not possible to achieve a comparable magnitude of turnover. With a similar saturation behavior, no significant further increase in activity above 4 μg CP per well and about half the ABTS turnover rates of sticks were observed. By contrast, stoichiometric amounts of pure biotinylated linkers produced the lowest substrate conversion rates in all cases, with no saturation reached in the concentration range appropriate for TMV scaffolds.

Whether elevated non-stoichiometric biotin linker amounts would support similar turnover rates as TMV sticks at their most efficient saturation concentration of 3.5 μg sticks (corresponding to 0.1 nmol biotin) per well was tested next. Indeed, 10–50 nmol biotin linkers applied directly, i.e., 100- to 500-fold excess over the linkers exposed on TMV, allowed for comparable ABTS conversion. It was not possible, though, to increase activities further by using more linker devoid of carrier templates: above 50 nmol of linkers per well activities declined again (Supplementary Figure 3).

Relative activities obtained with the same [SA]-enzyme input in the different immobilization layouts (Figure 1C) for saturated and non-saturated plate surfaces (Figures 3A,B) showed that the use of TMV_Cys_/Bio sticks as adapters for [SA]-enzyme immobilization yielded 30–45 times higher turnover rates, CP_Cys_/Bio a 15- to 25-fold, and directly applied maleimide-PEG-biotin linkers a 5–7-fold increase only (Figure 3B), if compared to the corresponding values for directly adsorbed enzymes without any carrier template. The low activities in plain microtiter plates for non-specific attachment of the [SA]-GOx and [SA]-HRP conjugates from their stock solutions reflect the competition between BSA and [SA]-enzyme molecules for the support's binding sites.

Taken together, the fashioned TMV nanosticks provide very efficient adapters for the immobilization of the commercial bi-enzyme glucose-sensing system in conventional high binding microtiter plates.

### Atomic force microscopy (AFM) analysis

**Figure 4:**
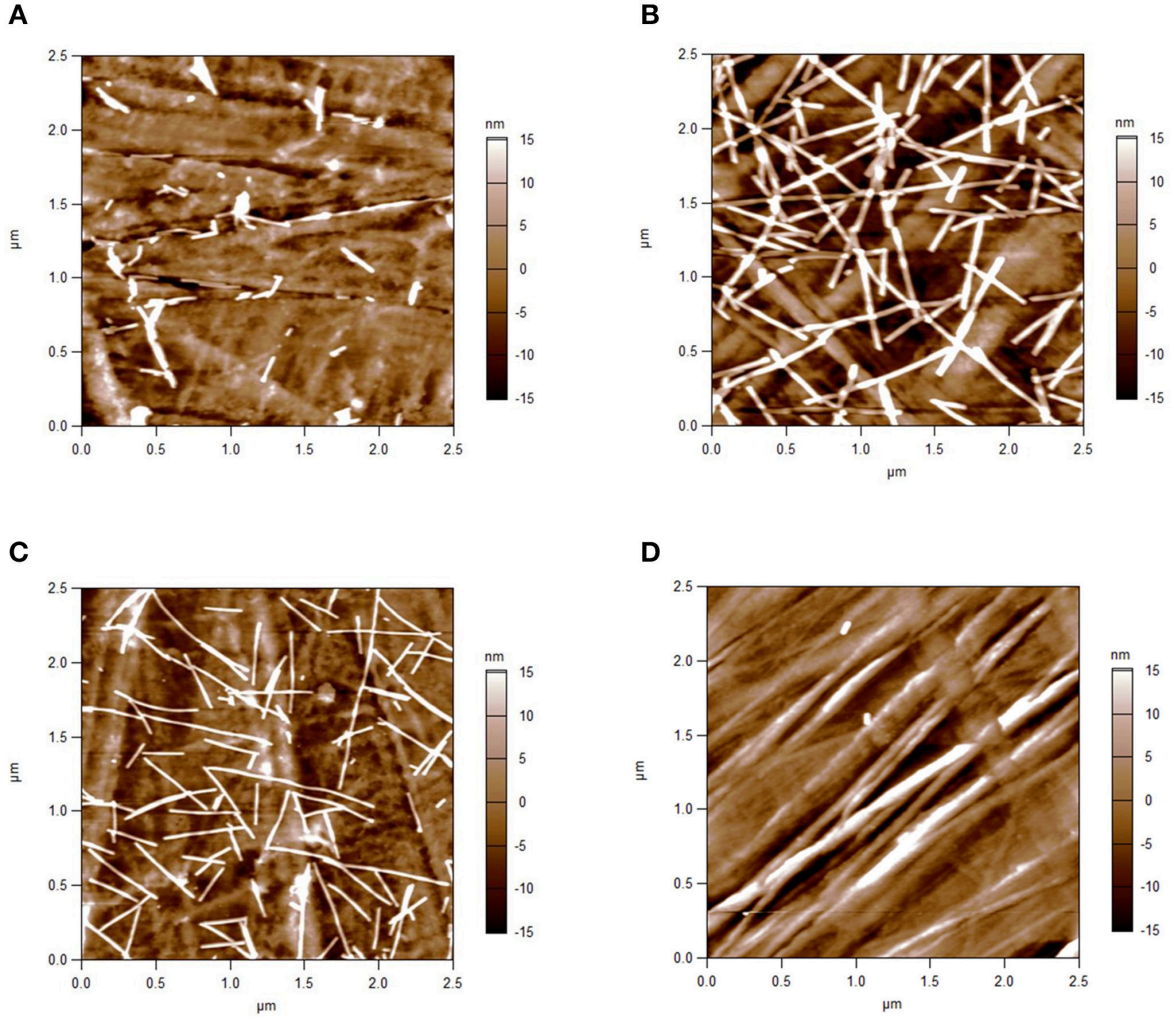
**AFM height images of TMV_**Cys**_/Bio sticks immobilized in microtiter plates**. Intermittent contact mode-AFM height images of TMV_Cys_/Bio nanosticks equipped with [SA]-enzymes after their immobilization on the bottom of high binding microtiter plate wells, and of a well subjected to direct binding of the enzymes. Well bottoms after application of **(A)** 1.5 μg adapter sticks: low packing density of TMV; **(B)** 3.5 μg; and **(C)** 5.5 μg sticks: equally tight packing of TMV sticks with local overlay and partial formation of superimposed layers. **(D)** Control: high binding plate bottom devoid of adapter templates, revealing striated surface structures of the polymer.

The arrangement of enzyme-loaded TMV_Cys_/Bio stick adapters on saturated microtiter plate supports was imaged by AFM. Following the enzyme assay, bottoms of the cavities were analyzed in intermittent contact mode. The TMV_Cys_/Bio nanotubes with a natural length of 300 nm and a diameter of 18 nm of the TMV core (see Figure 2C for comparison) plus a few nm of the linker-[SA]-enzyme coating were detected with the corresponding AFM height (Figure 4) for 1.5 μg applied in a non-saturated well, 3.5 μg at the threshold of saturation and 5.5 μg to ensure full saturation. Viral rods were well-recognized against the background structures of the polystyrene support and appeared randomly orientated and well-dispersed, with some linear head-to-tail oligomerization as typical of TMV particles, but no significant lateral aggregation. The AFM images showed a low packing density for 1.5 μg TMV sticks (Figure 4A), and an equally tight packing of TMV sticks after application of 3.5 μg (Figure 4B) and 5.5 μg (Figure 4C), with several overlap sites indicating a partial formation of multiple layers. This analysis confirmed saturation above 3.5 μg TMV per well. As controls, AFM images of high binding plate bottoms equipped with enzymes exposed on CP aggregates or devoid of any adapter (data not shown), and of untreated plates lacking any protein coating (Figure 4D) were recorded, all of which were essentially indistinguishable from each other and revealed the striated polymer surface only.

### Reusability and stability of the adapter-enzyme systems

A major advantage of immobilized enzymes over their freely suspended counterparts may lie in an increased reusability in serial applications and prolonged storage stability (Katchalski-Katzir, 1993; Mateo et al., 2007; Singh et al., 2013). Hence the performance of [SA]-GOx and [SA]-HRP displayed on TMV-based adapter scaffolds, biotin linkers or directly on the plates (“free”) was examined upon consecutive uses and after extended storage.

**Figure 5:**
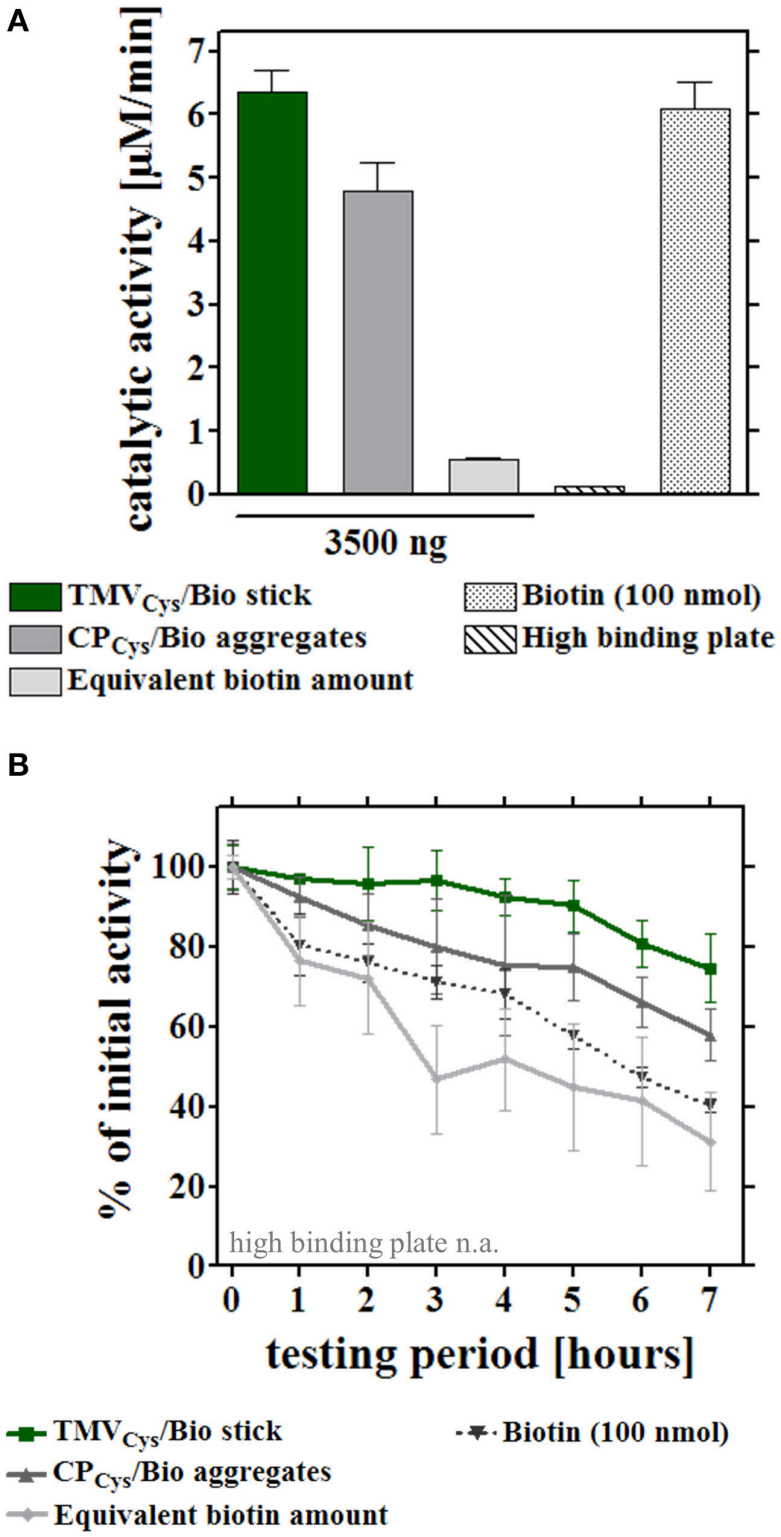
**Reusability of enzymes immobilized on different adapter templates**. Comparative analysis of enzyme activity upon serial hourly uses, after application of equal input amounts of enzyme mix to the different immobilized adapter templates or bare control wells. **(A)** Initial catalytic activities of the immobilized enzymes (input for all approaches 0.9 μg enzyme mix/well) on 3.5 μg TMV_Cys_/Bio sticks, 3.5 μg CP_Cys_/Bio aggregates, an equivalent amount of biotin linker, the microtiter support, or with 100 nmol biotin linker applied for increasing initial activity on those templates to the order of magnitude of TMV-based adapters (for details, refer to text). **(B)** Performance of the enzymes immobilized on the distinct adapters upon repeated use. Activities were determined for the same individual wells hourly over a period of 7 h in total. Initial catalytic activities of [SA]-enzymes on each adapter **(A)** were set to 100% (referential activities) and the percentages of remaining activities calculated.

**Figure 6:**
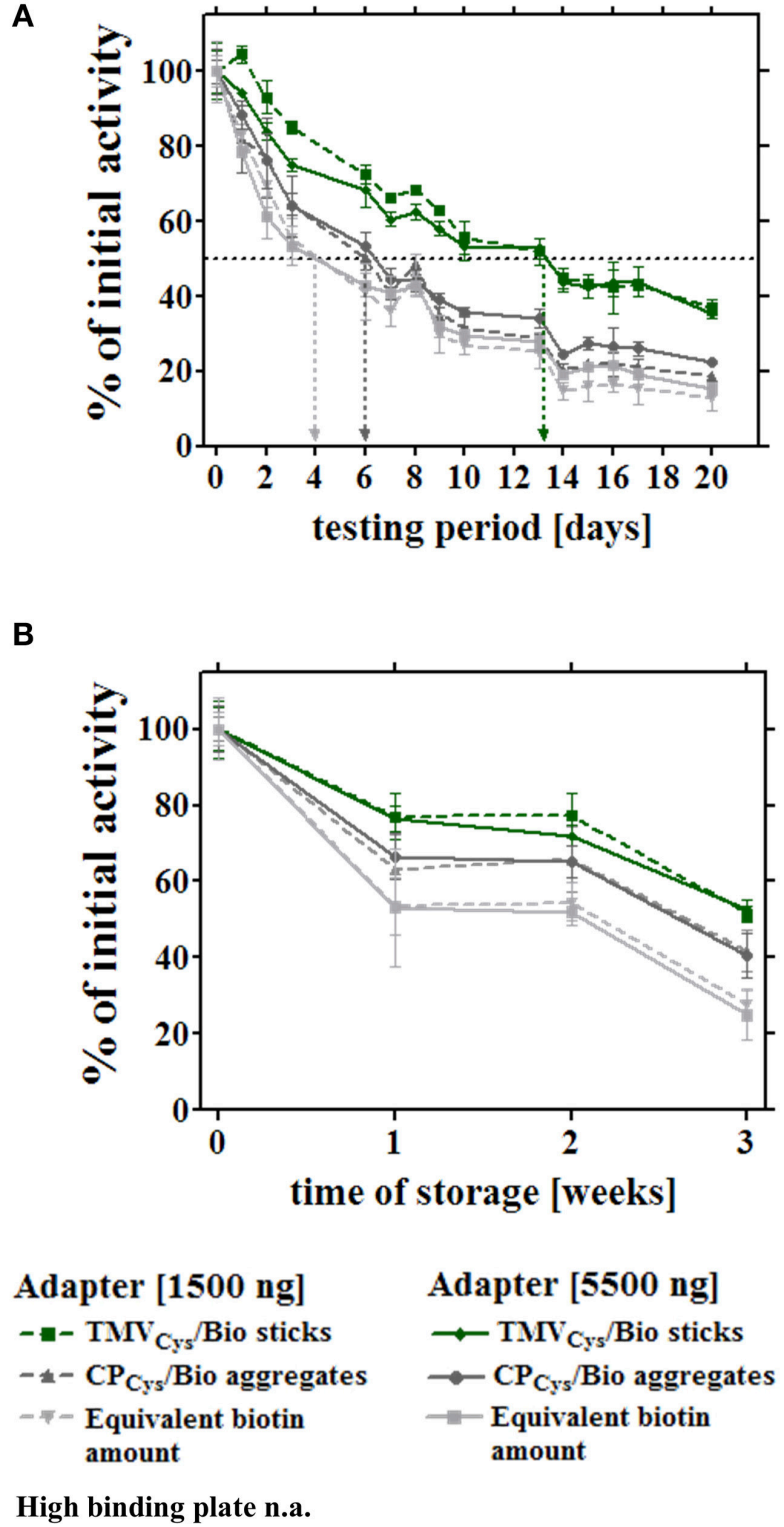
**Long-term reusability and storage stability of enzymes immobilized via distinct adapters**. Enzymatic activities were monitored through a period of 3 weeks for the different layouts described for Figure 5. Sample wells were either subjected to 14 consecutive uses **(A)** to determine the residual activities under these conditions, or prepared in parallel and stored idle up to the testing time point, as indicated **(B)**. **(A)** Immobilized enzymes were tested for their reusability and concomitant storage stability over 3 weeks, using 1.5 μg (non-saturated conditions) and 5.5 μg adapters (saturated conditions), or the corresponding linker molarity, according to the legend. Values determined for bare plates ranged below the level of detection from the second measurement on, and are not added to the diagram (n.a.). After the initial catalytic activities were determined, wells were washed with 1x PBS-T, stored in 10 mM SPP pH 7.0 at 4°C for at least 24 h, and incubated with substrate solution, washed and stored repeatedly, as indicated by the data points and legend. Arrows indicate half-lives of the distinct enzyme-adapter systems, corresponding to 50% of their referential initial activity. **(B)** Activity of enzymes exposed on different adapters as above, subjected to a first use at the different time points indicated, over a period of 3 weeks. For details, refer to text.

In a first set of experiments, the different enzyme-adapter systems were investigated for their reusability by repetitive testing. 3.5 μg TMV_Cys_/Bio sticks, CP_Cys_/Bio aggregates, and equivalent amounts of biotin linker as adapters, and directly bound enzymes were compared. In addition, test wells with high amounts (100 nmol) of biotin linkers were included, which yielded initial activities in the range of those achieved in the presence of the TMV sticks (Figure 5A). After immobilization as above, turnover rates were determined for the distinct layouts hourly over a period of 7 h, and the remaining activities were calculated after each consecutive use (Figure 5B). Notably, the [SA]-GOx/[SA]-HRP enzyme system on TMV_Cys_/Bio adapter sticks retained full activity for at least four cycles, before it slowly decreased and reached a residual activity of 75% after eight serial uses. The other samples showed a continuous decrease of turnover rates already after the first use, with residual activities of about 60% on CP_Cys_/Bio aggregates, 30% on equivalent amounts and 40% on highly concentrated biotin linkers. For directly immobilized enzymes, activities were below the level of detection (Figure 5B). In an extended time frame of 3 weeks and intermediate storage at 4°C, 14 measurements in total showed that after such a long-run application and all serial uses, the TMV_Cys_/Bio stick-scaffolded enzymes exhibited a remaining activity of around 50%, considerably superior to every other layout. Their half-life was increased to 13 days, while upon exposure on CP_Cys_/Bio aggregates this was reached already at day 6, i.e., with only a minor increase compared to day 4 detected for the enzyme system immobilized on biotin linkers directly applied to the plate wells (for details, refer to Figure 6A). These results indicate a remarkably stabilizing effect of the TMV_Cys_/Bio sticks on the enzymes' performance.

Second, the storage stability of the [SA]-GOx/[SA]-HRP mixture bound via different adapters or directly to plates was determined over a period of 3 weeks without intermittent use, applying 8-well strip plates. The samples were prepared simultaneously at time point zero, but measured for glucose detection activity after varying storage periods in buffer at 4°C (Figure 6B). The most pronounced loss of activity was observed during the first week of wet storage for all setups, with the least decline of conversion rates in the presence of TMV sticks. After 3 weeks, enzymes exposed on TMV_Cys_/Bio adapter sticks still retained 60% of the initial activity, whereas in layouts with CP_Cys_/Bio aggregates or maleimide-PEG-biotin linkers as adapters, the [SA]-GOx/[SA]-HRP system exhibited less than 45 or 30% activity, respectively. In the absence of adapter templates, remaining turnover rates were below the detection level.

In conclusion, all data obtained in the different assays on enzyme stability in distinct immobilization layouts consistently proved a preserving effect exerted by TMV nanosticks on enzymes linked to their surface.

## Discussion

Against the background of a broad literature on the use of TMV as building block of nanoscale materials with robust functionality, this study presents the proof-of-principle that it can serve as efficient biotemplate for the high-density immobilization of active enzymes on the viral surface. Beyond this, the strong stabilizing effect of biotinylated TMV sticks applied as adapters for streptavidin-conjugated enzymes in conventional microtiter plates is an important finding.

Bifunctional chemical cross-linkers were also used by Smith et al. (2006) who conjugated amine-reactive N-hydroxysuccinimide ester (NHS)-activated PEG_4_–biotin linker molecules to surface-exposed lysine residues on a TMV_Lys_ variant to couple green fluorescent protein (GFP)–[SA] fusion proteins. The conjugation reaction, however, yielded only about 20% modified CP subunits using a 24-fold linker excess (Smith et al., 2006). The results presented here (Figure 2A) indicate a superior reactivity of the maleimide-activated linkers with thiols, possibly through a better steric accessibility of the cysteine residue since the S3C is closer to the surface-exposed CP N-terminus (Geiger et al., 2013) than the K4 of the TMV CP variant addressed previously (Smith et al., 2006).

The coupling efficiency (Figure 2B) achieved with the maleimide-PEG_11_-biotin linkers led to the immobilization of about thousand enzyme molecules per TMV nanorod. This was more than a hundred times higher than the number of enzymes that could be installed on the outer surfaces of frequently applied spherical plant viral templates such as CPMV or CCMV (Wang et al., 2002; Aljabali et al., 2012; Putri et al., 2015), for which reason those platforms seem better suited as nanocage reactors to exploit the spatial confinement inside (Minten et al., 2011; Maity et al., 2015). In contrast, the immense surface increase enabled by elongated plant virus scaffolds, in combination with their multivalence on the nanoscale, is most attractive for concentrating and installing well-accessible functional biomolecules at sites of interest in different environments (as exemplified for tobamovirus rods in the introduction; reports on flexuous plant viruses comprise Uhde et al., 2005; Carette et al., 2007; Rioux et al., 2012; Pille et al., 2013; Shukla et al., 2013). To our knowledge, however, rod-shaped viruses have not been equipped with biological enzymes so far, and also among the filamentous virus-based biohybrids, examples for enzyme exposure are scarce (Carette et al., 2007; Pille et al., 2013). A frequent obstacle is caused by a low genetic tolerance of the virus genomes, which leads to backmutations, complete failure or low surface coverage after conventional genetic fusion, or inefficient chemical linkage of biological enzymes. This is why novel well-controllable genetic modification strategies and fresh ideas for versatile intermolecular coupling approaches are sought after (Cardinale et al., 2012).

**Figure 7:**
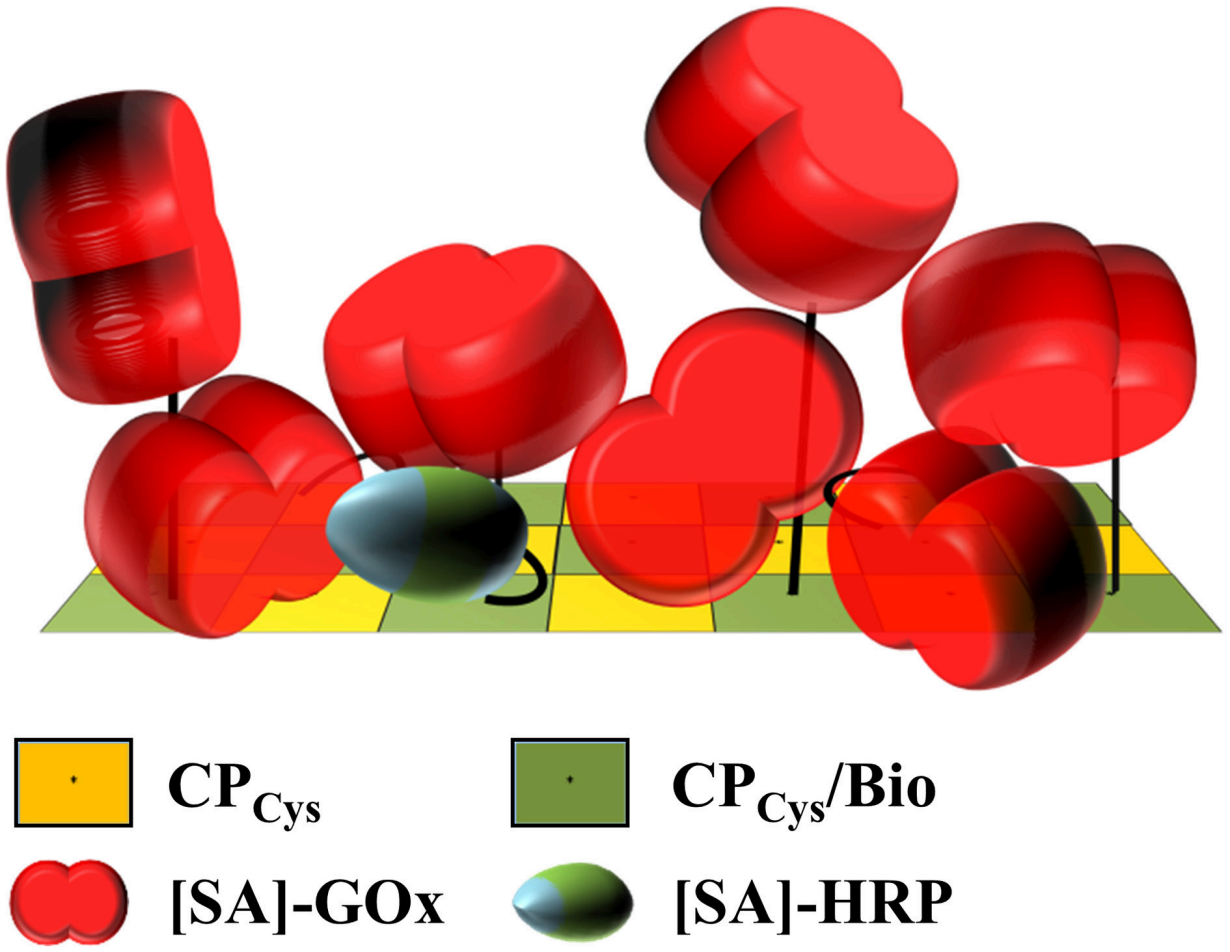
**Model of putative enzyme arrangement on a biotinylated surface segment of a TMV stick**. Schematic drawing demonstrating the putative arrangement of [SA]-GOx and [SA]-HRP installed on biotin linkers of 6 nm total length on the TMV surface, taking into account their molar ratio applied, the binding efficiencies achieved, the enzyme conjugates' dimensions and the volume generated by the maleimide-PEG_11_-biotin linkers above the TMV_Cys_ surface. Using a 22-fold excess of biotin linkers relative to the viral CP_Cys_ (yellow) molecules, every second CP_Cys_ was fashioned by a linker (CP_Cys_/Bio, green). Of those, virtually every biotin was shown to couple an [SA]-enzyme. This is likely to result in a staggered arrangement of the enzymes on the TMV surface. The flexible and 6 nm long PEG_11_ spacers as part of the linkers would therefore be essential to increase the surface volume of TMV accordingly. Refer to text and Supplementary Figure 5 for details.

Here, a two-step procedure based on the coupling of biotin cross-linkers and subsequently enzyme-streptavidin conjugates attained full occupation of all biotin linkers installed (Figures 2A,B), with a dense enzyme coverage of the viral nanosticks over their full length as confirmed by TEM (Figure 2C). A rough estimation of the volume occupied by the amount of enzymes evidently bound to the TMV surface (Supplementary Section 5) revealed that obviously, the linkers' flexible PEG_11_-spacer arms allowed the immobilization of more than a single layer, as it would be possible by a staggered arrangement (as proposed in Figure 7). The volume generated between the TMV surface and the end of fully extended linkers (≈133,000 nm^3^) can accommodate only ~500 [SA]-enzyme molecules out of the ~1000 found to bind. Therefore, the additional enzymes have to employ also the surface area above the linker-created volume, which provides space for another ~500 molecules. The assessment (Supplementary Figure 5) took into account the molecule sizes and probable shapes of GOx (Hecht et al., 1993) and HRP (Chattopadhyay and Mazumdar, 2000; Takahashi et al., 2000), respectively, and considered their conjugation to streptavidin homotetramers (Neish et al., 2002). Thus, the biotinylation efficiency of 50% was sufficient to reach the maximum surface occupation possible with linkers of 6 nm total length.

Immobilized enzymes may provide important advantages for both industrial and small-scale routine applications, such as reusability, improved shelf-life and stability against physical or chemical influences, easy handling and in some cases increased enzyme activity or specificity (Katchalski-Katzir, 1993; Mateo et al., 2007; Guisan, 2013; Singh et al., 2013). On the other hand, enzyme immobilization may go along with a reduction or even loss of activity resulting from sterically disadvantageous arrangements, unfavorable effects on their kinetic properties and limitations through mass transfer and non-specific interactions between reactants and immobilization matrices. An important and often underrated factor are the costs for carrier and fixing agents, or for the immobilization process itself (for a recent review with many industrial examples see Dicosimo et al., 2013). Hence procedures based on reasonably-priced templates supporting superior enzymatic performance are desired.

A broad spectrum of different immobilization techniques is reported in the literature, including adsorption, entrapment, cross-linking, covalent or affinity bonds as well as combinations thereof (Sassolas et al., 2012). While undirected immobilization is still most common at present, an oriented and strong binding of biologically active proteins may offer major advantages in that it can ensure good steric accessibilities of their active sites preserving full functionality, and increase stability. Primary prerequisite is the identification of suitable attachment points for specific interactions between the protein of interest and target sites on the carrier. Since techniques relying on the high affinity between biotin and avidin or streptavidin are widely used (Bayer and Wilchek, 1990; Turková, 1999; Singh et al., 2013), many well-optimized, cost-efficient reagents and compatible enzyme preparations are commercially available. In the field of immobilization supports, nanostructured materials of high surface-to-volume ratio have received special attention. They offer large surface areas and may exhibit physicochemical properties promoting robust enzyme activity. According to Kim et al. (2008) and Singh et al. (2013), an increase in the surface area of the supporting scaffold may result in an increased intrinsic and operational stability of the immobilized enzymes. In addition, the aquaphilicity of a scaffold is reported to influence the activity of enzymes (Reslow et al., 1988). Therefore, we suggest similar interactions based on physicochemical properties such as e.g., favorable hydrophilic/hydrophobic balance between the TMV nanostick surfaces, the immobilized enzymes and the aqueous solution as the surrounding medium, respectively, which might be responsible for the increased stability of the TMV/enzyme hybrid system compared to the other adapter/enzyme hybrid systems analyzed in this study. In combination with the higher immobilization rate compared to planar 2 D surfaces, such properties have resulted in increasingly frequent uses of nanostructured materials for a broadening spectrum of applications during the last decade (Kim et al., 2006; Ansari and Husain, 2012; Liu et al., 2012a).

As TMV sticks allowed efficient directed coupling of conventional enzyme conjugates, and are high-yield products readily accessible by greenhouse farming, they comply with all essential conditions of a technically promising biotemplate. We therefore decided for an in-depth characterization of the TMV-based adapter scaffolds, and analyzed the turnover rates obtained with free and immobilized enzymes. Full input activities were retained after coupling to TMV (Figure 2B). As opposed to some enzyme systems established in the confinement of virus-derived nanocontainers (Minten et al., 2011; Maity et al., 2015), educt conversion rates were not enhanced, thus no indication of substrate channeling on the TMV sticks was obtained.

These findings are in agreement with other studies on enzyme-decorated plant viral scaffolds. A similar GOx/HRP combination installed on the exterior of spherical CPMV capsids exhibited high retention of their activity even after sodium metaperiodate treatment, which generated reactive aldehyde groups on the enzymes amenable to chemical conjugation (Aljabali et al., 2012). Notwithstanding, the number of enzymes per virus was low (11 HRP and 2–3 GOx molecules on in total 120 small or large subunits). By contrast, cross-linker-mediated conjugation of a cysteine-engineered T4 lysozyme mutant to amines on the CPMV surface resulted in almost 100% coupling efficiency and above 80% activity maintained, in relation to that of unattached enzyme (Chatterji et al., 2004). Likewise, the filamentous *zucchini yellow mosaic virus* (ZYMV), genus Potyvirus, with about 700 nm length, was functionalized with 4-coumarate:CoA-ligase 2 on almost 90% of its ~2000 CP subunits, with full preservation of the enzymes' activity (Pille et al., 2013). This was achieved by a non-covalent, antibody-mediated “molecular sticker” sandwich assembly, and making use of enzymes fused to the immunoglobulin-binding peptide Z33 derived from staphylococcal protein A. The activity of *Candida* (now *Pseudozyma*) *antarctica* Lipase B (CALB), however, was reduced by a factor of about 45 upon covalent genetic fusion to *PVX*, in comparison to that of free CALB (Carette et al., 2007). FMDV 2a-guided expression led to a ratio of CALB–2A–CP to CP monomers in the virus particles of approximately 1:3. This concept circumvented the need of post-harvesting virus modification and remains to be evaluated for other enzyme species which might function with superior activities on PVX.

The distinct adapter types (TMV_Cys_/Bio sticks, CP_Cys_/Bio aggregates, and maleimide-PEG_11_-biotin linker molecules; Figure 1C) investigated here identified TMV sticks as the best choice. They achieved up to 45-fold substrate conversion rates and thus signal output, compared to an equal input of [SA]-GOx/[SA]-HRP directly applied to bare microtiter plate wells (Figure 3). CP aggregates will attach to the solid support in a largely undirected manner, for which reason only a subfraction of their biotin moieties may be expected to participate in enzyme binding. Their steric accessibility might be less favorable in general. For linker molecules, only a 100- to 500-fold excess allowed trapping of enzyme amounts enabling catalytic activities similar to those achieved on TMV backbones; a further increase was not possible probably due to full saturation of the microtiter plate wells. Taken together, TMV nanorods are likely to impart the largest increase in immobilization-competent surface (see also Figures 2C, 7).

Adequately immobilized enzymes may exhibit further advantages over freely suspended preparations, namely an increased shelf-life and/or superior reusability. Hence we investigated the influence of the different adapter scaffolds on the enzymes upon repeated uses and extended storage periods, respectively. TMV sticks consistently proved the best adapter material, with pronounced stabilizing effects on the GOx/HRP enzyme system in both layouts (Figures 5, 6). Stabilization of biomolecules through immobilization typically results from an advantageous combination of different factors: Particularly, linkage to a solid support may reduce the frequency of conformational changes, thereby increasing the durability of functional polypeptide chains (Mateo et al., 2007). Thus, various materials including inorganic or carbon nanotubes have been shown to stabilize immobilized catalytic activities (e.g., Ai et al., 2014; Azevedo et al., 2015). Efficacious molecules are also known to protect protein structures by lowering the surface tension of water and often serve as solvent additives in commercial formulations (Arakawa and Timasheff, 1983). Besides e.g., sugars, salt and glycerol (Arakawa and Timasheff, 1982, 1983), certain proteins such as BSA are most effective, with a clear correlation between their extent of surface hydrophobicity and the extent of stabiliziation achieved (Chang and Mahoney, 1995). Despite its negative net charge, the outer nanostructured TMV tube surface profile exhibits a repetitive pattern of both negative and positive patches at near-neutral pH, which are interspersed by hydrophobic domains (Wadu-Mesthrige et al., 1996; Bittner et al., 2009; Ge and Zhou, 2011). Obviously, this composition meets the physicochemical prerequisites (Singh et al., 2013) underlying reliable enzyme stabilization. Released CP subunits and aggregates thereof, with lateral and inner CP portions accessible, differ considerably in both amino acid composition and charge from the intact particle coat (Namba et al., 1989). The results of this study therefore suggest rod-shaped TMV assemblies to be highly efficient protein stabilizers, beyond being advantageous nanoadapter systems applicable on conventional microtiter plate supports.

## Conclusion

TMV_Cys_ nanotubes may be equipped with an ultradense shell of enzymes by means of thiol-reactive chemical biotin-cross-linkers and streptavidin conjugate capture. The two-enzyme system GOx/HRP displayed that way retained full activity, and could be immobilized in microtiter plates as the TMV scaffolds constituted efficient adapters for the functionalization of plate wells. Compared to related adapter templates, namely biotinylated TMV CP_Cys_ aggregates and the commercially available linker alone, TMV sticks exhibited superior performance. Not only did they allow for up to 45-fold enhanced catalytic activity in relation to that achieved with an equal enzyme input into bare plate cavities, they also came out to exert a surprisingly strong stabilizing effect. About 75% of the initial activity was still obtained upon the eighth hourly use, and 50 to 60% of the enzymes' activity persisted even through 3 weeks of wet storage, either idle or with 14 consecutive uses, respectively. Although TMV CP aggregates proved to be considerably advantageous over plain biotin linker scaffolds as well, rod-like TMV adapters consistently surpassed their efficiency. This might be attributed to an optimum combination of their outer surface characteristics and a sterically favorable shape.

## Outlook

Miniaturization technology and new hierarchically structured materials have reduced the dimensions of biosensors to the nanoscale, and concomitantly improved both their sensitivity and stability. Since the introduction of microfluidic devices (Manz et al., 1990) these systems have been substantially refined so that there is an increasing interest to use microchannels also for enzymatic applications. So far, a broad spectrum of polymers and inorganic materials like poly-methyl-methacrylate (PMMA) and polystyrene, but also glass and silicon have been tested for their ability to act as carrier supports for enzymes (Cerqueira et al., 2014). Recently a new trend is emerging toward the fabrication of biomimetic or bio-combined materials, since the remarkable precision and self-assembly properties of bio-derived materials provide considerable advantages (Pandey et al., 2008; Petrenko, 2008; Zhang et al., 2009; Singh, 2011).

**Figure 8:**
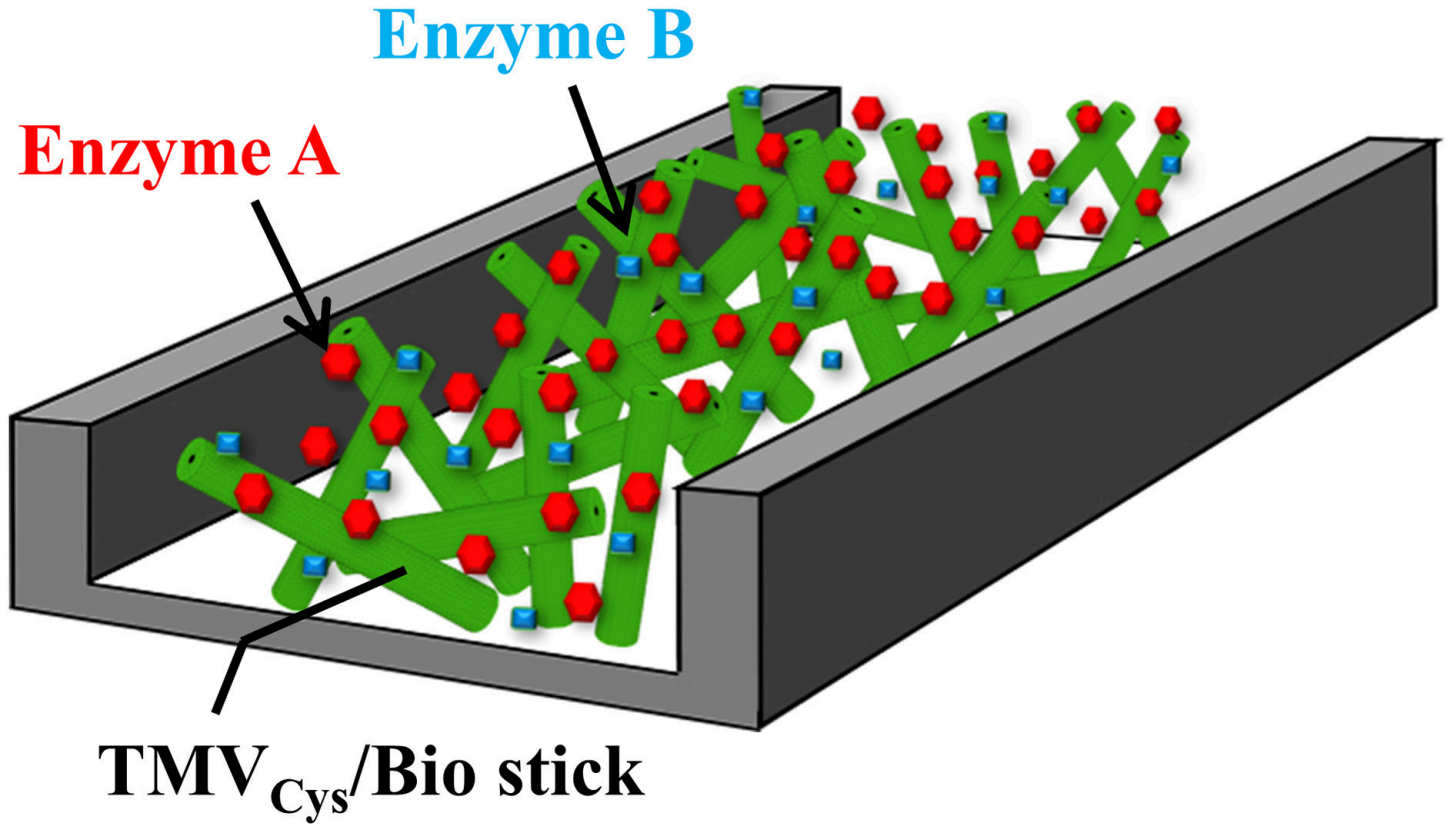
**Suggested use of TMV sticks as adapter matrix in a flow-cell**. Schematic drawing of a possible design of a flow-channel utilizing TMV_Cys_/Bio sticks for enzyme immobilization, e.g., for online monitoring purposes or lab-on-a-chip-based sensing. See text for details.

The enzyme-decorated TMV sticks may therefore find future uses not only in conventional assays, but might also render attractive biofunctional nanoparticle coatings for lab-on-a-chip devices and in-flow detectors (as depicted in Figure 8). Different TMV CP variants can be combined either in individual nanorods, or in blends of distinct TMV variants, and addressed selectively by simple conjugation methods (Mueller et al., 2010; Geiger et al., 2013; Eiben et al., 2014). With mixtures of differently addressable TMV adapters, or sets of TMV particles exposing such coupling groups in variable surface densities, or on series of adjacent longitudinal domains, respectively, it should be possible to realize also multienzyme systems with fine-tuned stoichiometric portions, intermolecular distances, and pre-defined spatial arrangements of the enzyme partners. This might even yield layouts enabling substrate channeling and thus enhanced enzyme activity, as demonstrated for other multivalent biomolecule-derived scaffolds (Müller and Niemeyer, 2008; Wang et al., 2009). Furthermore, it is possible to generate expanded TMV-deduced nanoarchitectures *in vitro*, namely tri- and tetrapods (Eber et al., 2015) and star-like structures (Eber et al., 2013), by using modified RNAs which may allow the construction of three-dimensional skeletons for utmost enlargement of the effective surfaces.

TMV is widespread also in the natural environment, non-pathogenic to animals, and humans, and can be harvested from plants in high yields. As evidenced in this study, enzymes installed on TMV derivatives may retain full activity after their immobilization; such hybrid particles are readily accessible via a selective and quantitative straightforward linker coupling chemistry. In conclusion, TMV-based starting materials offer realistic opportunities for a cost-efficient fabrication of versatile ultrahigh-surface nanoadapter systems. Since tubular TMV assemblies seem to inherently possess a strong preservative effect on the biocatalytic performance of enzymes over weeks, the respective hybrids might be most interesting for applications in enzyme-based detector devices or on small- to pilot-scale bioconversion platforms.

## Authors contributions

CK has carried out the majority of experiments and optimized their design to characterize the distinct enzyme-equipped adapters, has drafted the manuscript and ensured thorough editing. KW has compared enzyme activities in solution and on biotemplates. FE has developed the original detection layouts and enzyme immobilization strategies. PK has planned and conducted the AFM analyses. CA has optimized adapter binding to substrates and AFM. HG has developed the project concept jointly with CW, and has coordinated bio/inorganic interface modifications. SE has evolved analyses on enzyme performance, shelf life, and reusability, and has instructed data acquisition and interpretation. FG has integrated and guided parallel tests to ensure circumspect analyses, and has edited the manuscript in collaboration with CW. CW is responsible for the conceptual design of this study jointly with HG, has synchronized the distinct experiments on the fabrication and evaluation of enzyme-exposing viral scaffolds, wrote parts of, and edited the manuscript. All authors have contributed text sections or images, have been involved in editing, approved the final version and agree to be accountable for all aspects of the work in ensuring that questions related to the accuracy or integrity of any part of the work are appropriately investigated and resolved.

### Conflict of interest statement

The authors declare that the research was conducted in the absence of any commercial or financial relationships that could be construed as a potential conflict of interest.
