# Supplementary material for: Modified TMV Particles as Beneficial Scaffolds to Present Sensor Enzymes
Source: Front Plant Sci. 2015 Dec 24;6:1137. doi: 10.3389/fpls.2015.01137 (PMC4689848; doi:10.3389/fpls.2015.01137)
Supplement: Supplementary file 1 [file DataSheet1.PDF]

frontiers

Supplementary Material

Modified TMV particles as beneficial scaffolds to present sensor enzymes

Claudia Koch[1], Katrin Wabbel[1], Fabian J. Eber[1], Peter Krolla-Sidenstein[3], Carlos Azucena[3], Hartmut Gliemann[3], Sabine Eiben[1], Fania Geiger[2], Christina Wege[1]*

*** Correspondence:** Christina Wege: christina.wege@bio.uni-stuttgart.de

1. Removal of unbound biotin linker molecules via ultracentrifugation

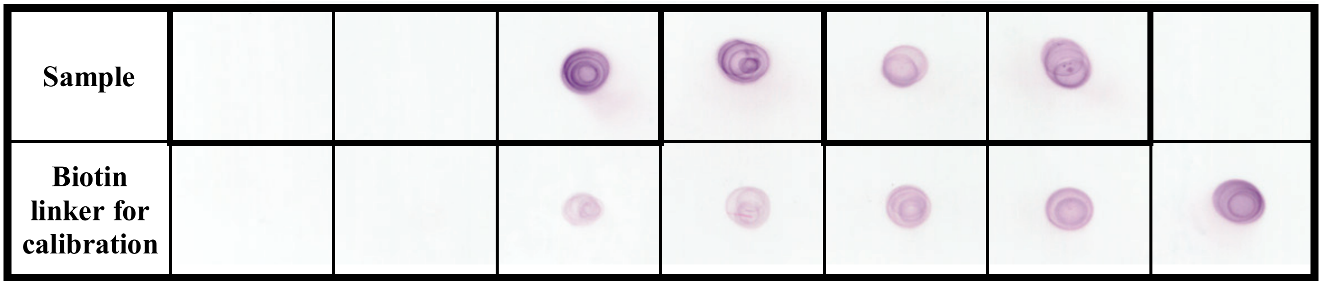

**Sample**	TMV$_{WT}$ (p - pure)	TMV$_{WT}$ (p - 1:10)	TMV$_{WT}$ (sn - 1:10)	Linker [input] (1:10)	TMV$_{Cys}$ (p - 1:10)	TMV$_{Cys}$ (sn - 1:10)	-
**Biotin linker [pmol]**	0	1	5	10	25	50	100

Supplementary Figure 1. Dot-blot analysis of ultracentrifugation (UC) approach for removal of unbound maleimide-PEG$_{11}$-biotin linker molecules (upper part: blot, lower part: legend).
To investigate if the UC procedure was suitable to separate unbound maleimide-PEG-biotin linker molecules from linker-modified TMV sticks, the following experiment was conducted: TMV$_{WT}$ or TMV$_{Cys}$, respectively, were incubated with maleimide-PEG$_{11}$-biotin linkers (with TMV$_{WT}$ used as control under equal conditions), as described in the material and methods section. Reaction mixes were subjected to UC, and pellets resuspended in SPP (see main article). Supernatants and pellets (pure and diluted 1:10 in 10 mM SPP pH 7.0) were spotted onto a nitrocellulose membrane and air-dried. The membrane was blocked with 1 % BSA in TBS-T (TBS, 0.1 % Tween-20) for 45 min, followed by three washing steps with TBS-T and incubation with streptavidin-conjugated alkaline phosphatase ([SA]-AP; Roche, Mannheim, Germany; 1:3000 in blocking solution) at RT for 45 min. After washing (6x5 min in TBS-T) and equilibration at pH 9.0, biotin linkers were detected by AP-mediated violet stain precipitated from an NBT/BCIP substrate solution. As expected, for TMV$_{Cys}$, signals were detected on both pellet and supernatant samples (right box), while for TMV$_{WT}$, no signal developed on pellet samples (pure or diluted; left box), indicating complete removal of unbound biotin linkers via UC.
Samples: fractions of the linker conjugation/UC procedure except for the 'Linker [input]' sample (equal input lacking TMV; diluted mix directly spotted without UC). **Biotin linker:** calibration series.
Abbreviations: p = pellet; sn = supernatant.

2. Specificity of enzyme immobilization via biotin-streptavidin binding

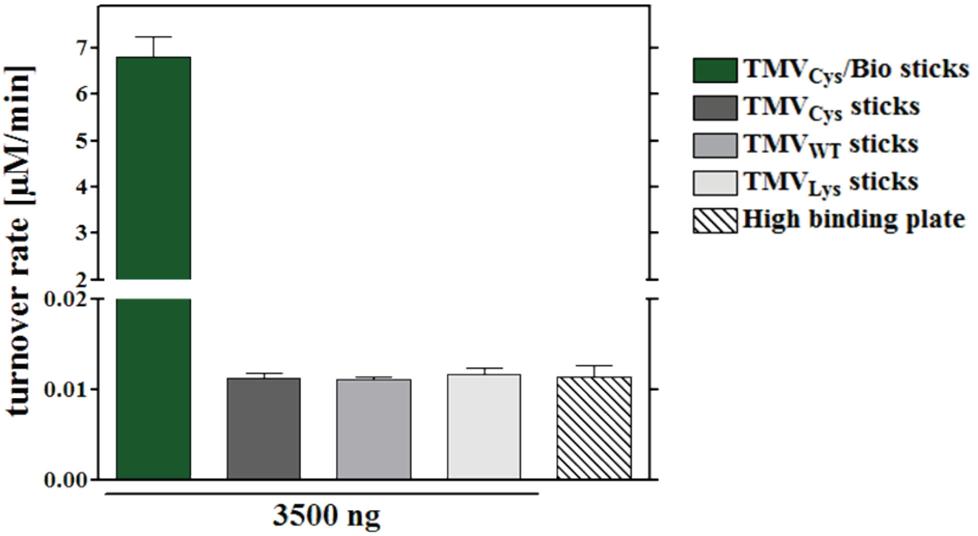

Supplementary Figure 2. Specificity of the biotin-streptavidin interaction underlying enzyme immobilization on the different adapter scaffolds.
To investigate differences between selective bioaffinity-mediated binding and putative non-specific adhesion of [SA]-enzymes to the TMV scaffolds, equal aliquots of non-biotinylated TMV particles exposing distinct reactive groups on their outer surfaces (TMV_{Cys}, TMV_{WT}, TMV_{Lys}) were immobilized in microtiter plate wells and incubated with equal amounts of the enzyme conjugates. Bare high binding plate wells were treated accordingly. Substrate conversion rates were determined and are shown in the histogram, as specified by the legend. In the presence of TMV_{Cys}/Bio nanotube-adapters, ABTS turnover rates of up to seven µM/min were obtained, while neither directly adsorbed [SA]-enzymes, nor the presence of bare TMV_{Cys}, TMV_{WT}, and TMV_{Lys} lead to significant enzyme activity detectable in the plate's wells. This indicates the biotin-streptavidin interaction to be crucial for a successful immobilization of [SA]-enzymes, and rules out their non-specific adsorption to the TMV CP surface.

3. High non-stoichiometric biotin linker amounts for capturing [SA]-enzymes

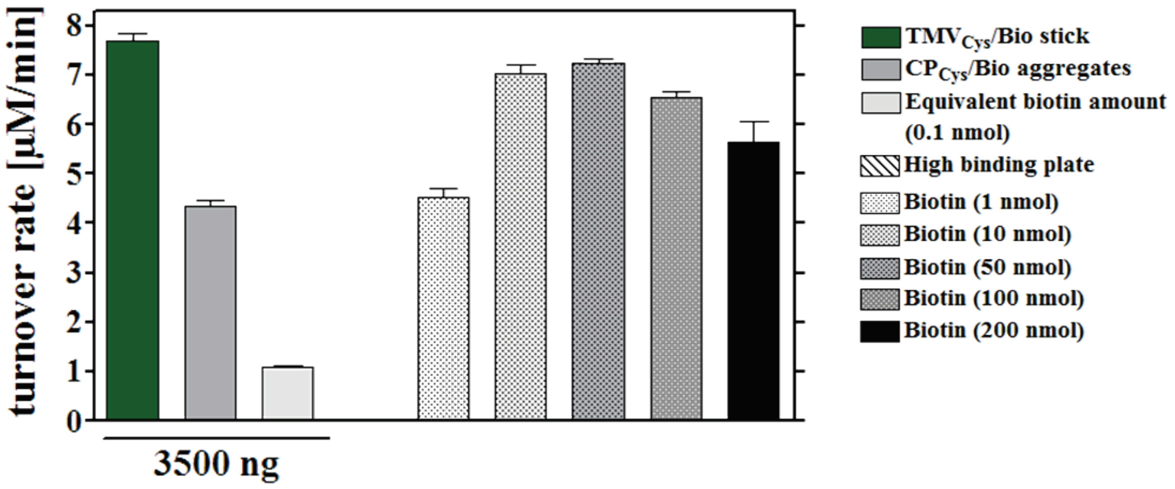

Supplementary Figure 3. [SA]-enzyme immobilization via high, non-stoichiometric biotin linker amounts applied to the microtiter plate supports.
We sought to find out if significantly increased, non-stoichiometric biotin linker amounts applied directly as adapters on the plate well support would allow substrate conversion rates similar to those obtained upon use of 3.5 µg TMV$_{Cys}$/Bio stick adapters. Therefore, biotin linker amounts between 0.1 nmol (corresponding the linker amount accessible on 3.5 µg TMV$_{Cys}$/Bio sticks) and 200 nmol per well were employed. Using 10-50 nmol per well (which is equivalent to a 100- to 500-fold excess over the linker input presented on 3.5 µg TMV$_{Cys}$/Bio at the biotinylation efficiency of 50 % used here), it was indeed possible to reach virtually the same turnover rates. Interestingly, above 50 nmol biotin linkers per well, turnover rates declined again, probably as a result of steric hindrance on plates with more than a single linker layer attached.

4. Enzyme kinetics: Determination of [SA]-GOx and [SA]-HRP activity

Initially, the individual enzymatic activities of [SA]-HRP and [SA]-GOx, respectively, were determined using H_2O_2 or/and glucose in combination with ABTS as substrates, as follows.

[SA]-HRP activity: method.
[SA]-HRP activity was determined using mixtures of 25 µg [SA]-HRP in 100 µl 100 mM NaOAc pH 5.1 and 50 µl 20 mM ABTS. Reactions were started by adding 50 µl H_2O_2 of different concentrations (final concentrations [f.c.] 0.03 mM to 21.87 mM). The turnover of ABTS into the corresponding radical (ABTS*) was monitored spectrophotometrically at 405 nm. Absorption values were converted to corresponding concentrations using the Lambert-Beer Law (for absorption coefficient and specific experimental conditions see materials and methods section 3.3), and plotted against time. Reaction rates v were determined from the slopes of the linear sections.

The resulting data were analyzed based on the Michaelis–Menten theory (1) and the Lineweaver-Burk transformation (2).

$$v = \frac{v_{max} \cdot [S]}{K_M + [S]} \qquad (1)$$

$$\frac{1}{v} = \frac{K_M + [S]}{v_{max} \cdot [S]} = \frac{K_M}{v_{max}} \cdot \frac{1}{[S]} + \frac{1}{v_{max}} \qquad (2)$$

v (reaction rates) were determined experimentally, K_M and v_{max} obtained by nonlinear curve fitting according to Lineweaver-Burk. k_{cat} was calculated with equation (3).The Supplementary Table 1 lists the mean values of these kinetic parameters from four independent experiments (see below).

$$k_{cat} = \frac{v_{max}}{[E]_0} \qquad (3)$$

The catalytic efficiencies of the systems were calculated as k_{cat}/K_M from the mean values.
[S] represents the concentration of the substrate, $[E]_0$ the total amount of enzymes used, and v the reaction rate. v_{max} specifies the maximum reaction rate of the system, whereas K_M is defined as the substrate concentration at $v_{max}/2$. k_{cat} gives the turnover number of the enzymes, and k_{cat}/K_M is the resulting constant that is a measure for catalytic efficiency.

[SA]-GOx activity: method.
The kinetic parameters of [SA]-GOx were obtained using a kinetic procedure adapted from Duley and Holmes [1]. This assay involves the coupling of the H_2O_2 production from [SA]-GOx to the reduction of ABTS by [SA]-HRP, resulting in the spectrophotometric measurement of the H_2O_2 production by monitoring the formation of $ABTS^*$ at 405 nm. The reaction rates for different glucose concentrations were determined at RT using mixtures of 6.25 µg/l [SA]-HRP, 200 µg/l [SA]-GOx, 50 mM NaOAc pH 5.1, 5 mM ABTS and 1 to 50 mM glucose (f.c.). Activity constants were estimated on the basis of the Michaelis–Menten theory and the Lineweaver-Burk transformation using the mean values of three independent experiments (as described above).

Kinetics of free [SA]-GOx and free [SA]-HRP: results.
In the Supplementary Figure 4, A-B show the hyperbolic curves obtained after plotting v against the substrate concentrations [S], and the double reciprocal Lineweaver-Burk plots of the experimental data. The resulting Michaelis–Menten parameters and the catalytic efficiencies (k_{cat}/K_M) for [SA]-HRP and [SA]-GOx, respectively, are shown in the Supplementary Table 1.

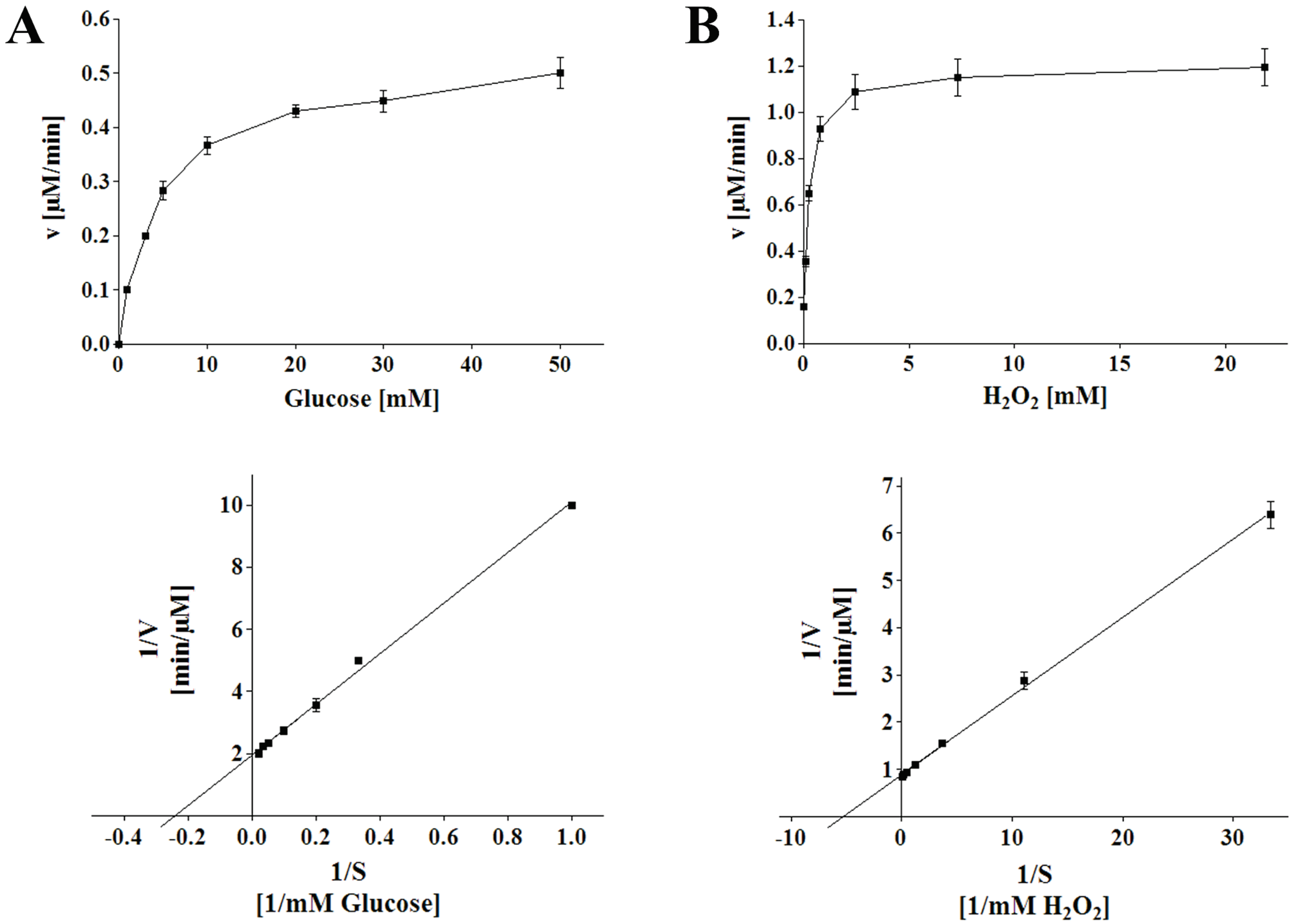

Supplementary Figure 4. Michalis-Menten and Lineweaver-Burk plots for determination of the catalytic constants of [SA]-GOx (A) and [SA]-HRP (B).
Upper row: Michaelis-Menten curve fittings after plotting the reaction rates *v* against the substrate concentrations [S], referring to (A) glucose and (B) H_2O_2 as described in the text. Lower row: corresponding Lineweaver-Burk plots.

Supplementary Table 1. Kinetic parameters determined for [SA]-GOx and [SA]-HRP, respectively (mean values retrieved from three or four experiments each, see text).

	**[SA]-GOx**	**[SA]-HRP**
V_{max} [µM/min]	0.51	1.13
K_M [µM]	4140	190
k_{cat} [1/s]	9.33	157.68
k_{cat}/K_M [1/(µM·s)]	0.002	0.840

5. Model calculations for the putative enzyme arrangement on TMV

Volumes and surface area

	Volume [nm^3]	Surface [nm^2]
a: TMV_{Cys}	≈ 76,300	≈ 17,000 (w/o ends of cylinder)
b: TMV_{Cys}/Bio	≈ 209,500	≈ 28,000 (w/o ends of cylinder)
c: Linker-generated	≈ 133,000	

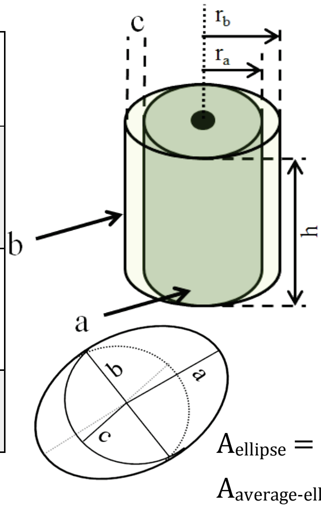

$V_a = \pi r_a^2 h$

$V_b = \pi r_b^2 h$

$V_c = b - a$

$A = 2 \pi r h$

h = 300 nm
r_a = 9 nm
$r_b = (r_a + \text{linker})$
Linker length: 59.1Å

$$A_{ellipse} = \pi \frac{a}{2} \frac{b}{2}$$

$$A_{average\text{-}ellipsoid} = (A_{ab} + A_{ac} + A_{bc})/3$$

$$V_{ellipsoid} = \frac{4}{3} \pi \frac{a}{2} \frac{b}{2} \frac{c}{2}$$

Enzyme dimensions

Enzyme/Protein	MW [kDa]	Size (a b c) [Å]	Protein volume [nm^3]	[SA]-Enzyme volume [nm^3]	Protein average area [nm^2]	[SA]-Enzyme av. area [nm^2]
GOx[2]	160	70 x 55 x 80	161	270	36	58
HRP	44[3]	64 x 37 x 43[4]	53	162	18	40
Streptavidin [SA]	60	20.4 x 113 x 45[5]	109[4]		22	

Enzymes fitting on TMV_{Cys}/Bio

Enzyme mix	Enzymes fitting in volume c (133,000 nm^3)	Enzymes fitting on surface of cylinder b (28,000 nm^2)
GOx:HRP	~ 500	~ 490

Biotinylation efficiency: 50 %
→ 2130 CP/2 → 1065 CP_{Bio} → ~ 1000 enzymes per TMV_{Cys}/Bio
Molar ratio of GOx:HRP = 14:1
→ 1000 enzymes contain 934 GOx + 66 HRP molecules

Molar ratio of GOx:HRP = 14:1
Mean [SA]-enzyme volume:
(14 x 270 nm^3 + 162 nm^3) / 15 ≈ 263 nm^3
Mean [SA]-enzyme area:
(14 x 58 nm^2 + 40 nm^2) / 15 = 57 nm^2

Supplementary Figure 5. Rough assessment of enzyme dimensions and volumes/areas on TMV sticks. To delimit the possible arrangement of enzymes linked to every second CP (CP_{Bio}), the average volume of the enzyme molecules, the volume between the TMV_{Cys} surface and the linker ends (stretched conformation), and the surface area above this volume were calculated. GOx: a homodimer of ellipsoidal shape, 160 kDa[1]; HRP: a monomer with elongated shape[3], 44 kDa [2]; streptavidin: a homotetramer of 60 kDa[4] and a rough mean molecular volume of 109 nm^3 (mean volume adapted from [4]), linked in equal or non-stoichiometric oligomeric amounts to the enzymes. For the calculation, 1:1 [SA]:enzyme conjugation was assumed. Through the 5.9 nm long PEG-biotin linkers, a volume of ≈133,000 nm^3 between the TMV_{Cys} surface (r_a) and the linker ends in extended (stretched) conformation is generated. This volume would allow the immobilization of ~500 [SA]-enzymes per TMV stick. Approximately 490 [SA]-enzymes can find place above this first enzyme shell due to the PEG_{11}-spacer of the linkers, resulting in a staggered arrangement (as depicted in Figure 7).

6. Supplementary references

[1] Duley, J. and R.S. Holmes, *A spectrophotometric procedure for determining the activity of various rat tissue oxidases.* Anal Biochem, 1975. **69**(1): p. 164-9.

[2] Hecht, H.J., et al., *The 3D structure of glucose oxidase from aspergillus niger. Implications for the use of GOD as a biosensor enzyme.* Biosens Bioelectron, 1993. **8**(3-4): p. 197-203.

[3] Chattopadhyay, K. and S. Mazumdar, *Structural and conformational stability of horseradish peroxidase: Effect of temperature and pH.* Biochemistry, 2000. **39**(1): p. 263-270.

[4] Takahashi, H., et al., *Catalytic Activity in Organic Solvents and Stability of Immobilized Enzymes Depend on the Pore Size and Surface Characteristics of Mesoporous Silica.* Chem. Mater., 2000. **12**(10): p. 3301-3305.

[5] Neish, C.S., et al., *Direct visualization of ligand-protein interactions using atomic force microscopy.* British Journal of Pharmacology, 2002. **135**(8): p. 1943-1950.
